# Genomic Insights Into the Genetic Structure and Natural Selection of Mongolians

**DOI:** 10.3389/fgene.2021.735786

**Published:** 2021-12-08

**Authors:** Xiaomin Yang, Sarengaowa, Guanglin He, Jianxin Guo, Kongyang Zhu, Hao Ma, Jing Zhao, Meiqing Yang, Jing Chen, Xianpeng Zhang, Le Tao, Yilan Liu, Xiu-Fang Zhang, Chuan-Chao Wang

**Affiliations:** ^1^ Department of Anthropology and Ethnology, Institute of Anthropology, National Institute for Data Science in Health and Medicine, School of Sociology and Anthropology, Xiamen University, Xiamen, China; ^2^ State Key Laboratory of Cellular Stress Biology, School of Life Sciences, Xiamen University, Xiamen, China; ^3^ State Key Laboratory of Marine Environmental Science, Xiamen University, Xiamen, China; ^4^ Department of Forensic Medicine, Guizhou Medical University, Guiyang, China; ^5^ Institute of Biological Anthropology, Jinzhou Medical University, Liaoning, China; ^6^ Department of Pediatrics, Xiang’an Hospital of Xiamen University, Xiamen, China

**Keywords:** Mongolian, genetic heterogeneity, admixture history, natural selection, functional genes

## Abstract

Mongolians dwell at the Eastern Eurasian Steppe, where is the agriculture and pasture interlaced area, practice pastoral subsistence strategies for generations, and have their own complex genetic formation history. There is evidence that the eastward expansion of Western Steppe herders transformed the lifestyle of post-Bronze Age Mongolia Plateau populations and brought gene flow into the gene pool of Eastern Eurasians. Here, we reported genome-wide data for 42 individuals from the Inner Mongolia Autonomous Region of North China. We observed that our studied Mongolians were structured into three distinct genetic clusters possessing different genetic affinity with previous studied Inner Mongolians and Mongols and various Eastern and Western Eurasian ancestries: two subgroups harbored dominant Eastern Eurasian ancestry from Neolithic millet farmers of Yellow River Basin; another subgroup derived Eastern Eurasian ancestry primarily from Neolithic hunter-gatherers of North Asia. Besides, three-way/four-way qpAdm admixture models revealed that both north and southern Western Eurasian ancestry related to the Western Steppe herders and Iranian farmers contributed to the genetic materials into modern Mongolians. ALDER-based admixture coefficient and haplotype-based GLOBETROTTER demonstrated that the former western ancestry detected in modern Mongolian could be recently traced back to a historic period in accordance with the historical record about the westward expansion of the Mongol empire. Furthermore, the natural selection analysis of Mongolians showed that the Major Histocompatibility Complex (MHC) region underwent significantly positive selective sweeps. The functional genes, alcohol dehydrogenase (*ADH*) and lactase persistence (*LCT*), were not identified, while the higher/lower frequencies of derived mutations were strongly correlated with the genetic affinity to East Asian/Western Eurasian populations. Our attested complex population movement and admixture in the agriculture and pasture interlaced area played an important role in the formation of modern Mongolians.

## Introduction

The vast Eurasian steppe zone stretching from Hungary in the west to Mongolia and northeastern China in the east has witnessed a dynamic demographic history. Ancient DNA findings from Western Eurasian Steppe showed the massive continental-scale steppe population migrations, admixture, and turnover since the Early Bronze Age (Allentoft et al., 2015; Mathieson et al., 2015; Damgaard et al., 2018; Wang et al., 2019). Both archaeologically and genetically attested evidence also showed the Western Steppe populations migrated to the Eastern Steppe zone and had influenced the genetic makeup of the Eastern Eurasians (Damgaard et al., 2018; de Barros Damgaard et al., 2018; Narasimhan et al., 2019; Ning et al., 2019; Jeong et al., 2020; Wang et al., 2021), whose genetic structure with a west-east admixture cline of the ancestry of Ancient North Eurasian (ANE) and Ancient Northeast Asian (ANA) stretching from Botai in Central Asia to Lake Baikal, Mongolia, and Devil’s Gate Cave of Eastern Eurasian has existed during the Pre-Bronze Age periods (Siska et al., 2017; de Barros Damgaard et al., 2018; Jeong et al., 2020). The Eastern Steppe has served as a crossroad for human population movements and plays a pivotal role in achieving cultural exchanges. The eastward expansions of Western Steppe populations associated with the Yamnaya (ca. 3300–2700 BCE) and Afanasievo (ca. 3300–2500 BCE) cultures in the Early and Middle Bronze Age and later ones associated with Andronovo (ca. 1800–1300 BCE) and Sintashta (ca. 2200–1700 BCE) in the Late Bronze Age not only brought related culture into the Eastern Steppe but also substantially contributed to the gene pool of the Eastern Steppe, forming the genetic heterogeneity with west-east admixture cline of Western Steppe-related ancestry. An additional genetic influx related to Central/Southern Asia populations was detected in the Early Iron Age western Mongolia ancient populations, which still exists in modern Mongolic and Turkic speaking groups (Jeong et al., 2019; Jeong et al., 2020). Subsequently, Xiongnu (209 BCE–98 CE), the first historically documented empire founded by pastoralists, received more complex gene flows in accordance with the historical records and showed highly heterogeneous populations structure, harboring different Han-related ancestry and more recent Western Steppe-related ancestry (Damgaard et al., 2018; Jeong et al., 2020; Wang et al., 2021). The Mongol empire emerged and established the largest continental empire across Asia and eastern Europe in the 13th century, controlling vast territories and trade routes, and diverse populations flowed into the steppe heartland. However, the genetic heterogeneity of the Eastern Steppe during this period was lower than that of previous nomadic regimes, with more Eastern Eurasian-related ancestry, marking the beginning of the formation of the modern Mongolians’ gene pool (Jeong et al., 2020; Wang et al., 2021). Even though the Western Steppe-related ancestry fluctuated in ancient Mongolia populations, modern Mongolian groups still show some extent of affinity with Western Eurasian-related populations and show genetic structure with different proportions of the Western Eurasian-related ancestry (Bai et al., 2018; He et al., 2019; Jeong et al., 2019; Zhao et al., 2020).

Across the Eurasian Steppe, dairy is a staple food and traditional diet. At the beginning of the Bronze Age, the multi-phased introduction of pastoralism drastically changed lifeways and subsistence on the Eastern Steppe (Jeong et al., 2018; Wilkin et al., 2020). Milk consumption in Mongolia before 2500 BCE by individuals affiliated with the Afanasievo, Chemurchek (2750–1900 BCE) and the Deer Stone-Khirigsuur Complex (DSKC) cultures in Khövsgöl was confirmed by large-scale paleogenomics studies. In contrast, the whole genome analysis of ancient populations in Mongolia revealed that despite the pastoralist lifestyle with evidence of milk consumption, the absence of positive selection of lactase persistence-related gene (*LCT/MCM6*) leading to the negligibly low frequency of derived mutations conferring lactase persistence indicates that animal husbandry for livelihood was adopted in the Eastern Steppe by local hunter-gatherers instead of causing by massive populations movements and turnover in Mongolian (Jeong et al., 2018; Jeong et al., 2020).

Inner Mongolia Autonomous Region, located in northern China, adjacent to the Central Plain and the West Liao River in northeastern East Asia, and some parts of it belong to the Yellow River Basin, which is the cradle of millet farming of China, the Middle Neolithic Miaozigou Culture in Inner Mongolia showed the characteristic of northward expansion of millet farmers in the Yellow River Basin (Ning et al., 2020). Moreover, Inner Mongolia Autonomous Region has been a farming-pastoral transitional zone in East Asia since the development of agriculture in the Neolithic Age and served as a key communication point between the nomadic culture of the northern grassland and the farming culture of the Central Plain. In addition, south-north bidirectional migration and coastal route of population movement between East Asia and Siberia have impacted the observed genetic variations among modern East Asians (Yang et al., 2020; Wang et al., 2021). Here, we obtained high-density SNP data of 42 Mongolian individuals from the boundary between Inner Mongolia Autonomous of China and Mongolia to provide a dense portrait of the genetic structure of Mongolians. We aimed to address the following three questions: 1) the extent of genetic heterogeneity or homogeneity among geographically different Mongolians; 2) the admixture sources and timing of Mongolians; 3) the signals of natural selection and the environmentally adapted gene in Mongolians.

## Materials and Methods

### Sample Collections

We collected saliva samples from 42 Mongolian individuals from Baotou city of Inner Mongolia Autonomous Region. Each included individual followed the criteria of sampling collection that require people to have long-term resident history and do not have recorded intermarriages with other surrounding populations for at least three generations. Our work was approved by the Medical Ethics Committee of Xiamen University (Approval Number: XDYX2019009). Informed consent was obtained from all participants included in the study.

### Genotyping and Data Merging

Genotyping was performed on the Illumina arrays covering genome-wide 600,000 SNPs designed to identify all known paternal Y chromosome and maternal mtDNA lineages. We first analyzed the relatedness of individuals measured by IBD (identified by descent) segments using KING software (Manichaikul et al., 2010); unrelated individuals were identified using the value of kinship < 0.0442. A total of 39 unrelated participants without family relationships were retained for subsequent analysis. We conducted quality control using PLINK (Chang et al., 2015) with --geno 0.2, --hwe 10e-10, filtering 670,269 SNPs. Then, the whole genome data of Mongolian was merged with the availably published dataset, including the Genome-Wide Human Origins Array genotype dataset and ancient/modern DNA of China and ancient Eastern Eurasian samples from 1240K capture dataset from David Reich Lab (Damgaard et al., 2018; de Barros Damgaard et al., 2018; Narasimhan et al., 2019; Ning et al., 2019; Ning et al., 2020; Yang et al., 2020; Wang et al., 2021), generating a combined Human Origins (HO) dataset covering 72,037 SNPs for subsequent analysis. Apart from this, the 1240K capture dataset, just combining the 1240K dataset, covered 186,187 SNPs.

### Analysis of Population Structure and Relationships

We performed principal component analysis (PCA) on the merged dataset using the smartpca built-in EIGENSOFT package (Patterson et al., 2006). Modern individuals were used to calculate PCs, and ancient individuals were projected onto the pre-calculated components using the ‘‘lsqproject: YES’’ option. To characterize population structure further, we calculated *f*_*3*_ in the form of *f*_*3*_ (*population1*, *population2*; *Mbuti*) and *f*_*4*_ statistics using qp3Pop and qpDstat in the ADMIXTOOLS package (Patterson et al., 2012). We added the ‘‘f4mode: YES’’ option to the parameter file for calculating *f*_*4*_ statistics. We also estimated pairwise genetic distance by Fst using the smartpca program of EIGENSOFT (Patterson et al., 2006) with fstonly: YES and inbreed: YES parameter. We estimated relative genetic drifts and inferred a rooted maximum likelihood tree by TreeMix software (Pickrell and Pritchard, 2012). We conducted the best qpGraph-based models with population split and admixture events via the ADMIXTOOLS package.

### Analysis of Population Admixture History Based on Sharing Allele Frequency

To investigate ancestry components in our Mongolian sample compared with other published Mongolian studies in different regions, an unsupervised clustering approach implemented in ADMIXTURE (Alexander et al., 2009) was firstly conducted, after filtering linkage disequilibrium using PLINK (Chang et al., 2015) with “--indep-pairwise 200 25 0.4” option, which retained a total 61,866 SNPs. Ancestry components and cluster memberships of 2084 individuals from 189 ancient and modern populations were calculated using the ADMIXTURE software. Clustering was performed for K = 2 to K = 20 in 100 bootstraps with different random seeds; we calculated the cross-validation errors to choose the best-fitted model. We also conducted admixture-*f*_*3*_-statistics in the form *f*_*3*_(*Source1*, *Source2*; *Mongolian_sub*) using the qp3pop program with default parameters in ADMIXTOOLS to explore the potential admixture surrogates showing significantly negative *f*_*3*_ value. For modeling *f*_*4*_ statistics-based admixture and estimating ancestral proportions in Mongolian, we applied *qpWave* (Patterson et al., 2012; Haak et al., 2015; Agranat-Tamir et al., 2020) to test for variation in ancestry proportions among the Mongolian and other modern Mongolian-related populations and detect the minimum number of ancestral sources; *qpWave* tests whether each possible pair of groups (Test i, Test j) is consistent with being a clade—since separation from the ancestors of a set of outgroup populations. qpAdm (Patterson et al., 2012) was used to calculate target populations as a combination of ancestry proportions from putatively selected source populations (references). To evaluate potential sex bias, we applied qpAdm to both the autosomes (default setting) and the X chromosome (adding ‘‘chrom:23” to the parameter file) for comparing the difference in the estimated ancestry proportions. For a certain ancestry, we calculated sex bias Z-score using the proportion difference between P_A_ and P_X_ divided by their standard errors (Z=(P_A_-P_X_)/√ἀ_A_^2^+ ἀ _X_^2^, where ἀ_A_ and ἀ _X_ are the corresponding jackknife standard errors) (Mathieson et al., 2018). Therefore, a positive Z-score suggests that autosomes harbor a certain ancestry more than X chromosomes, indicating male-driven admixture, whereas a negative Z-score suggests female-driven admixture (Jeong et al., 2020). To understand the time scale of population mixture events in the Mongolian population, we used ALDER based on weight linkage disequilibrium statistics to date the admixtures with 28 years as one generation (Loh et al., 2013).

### Fine-Scale Genetic Structure Based on FineSTRUCTURE

Bayesian clustering implemented in FineSTRUCTURE was used to reconstruct polygenetic relationships and further identify population structure. To reduce the computational burden, we randomly sampled 10 to 20 individuals in a large reference group. We first phased genome-wide dense SNP data using the SHAPEIT2 version (Delaneau et al., 2013) and then conducted FineSTRUCTURE (Lawson et al., 2012) analysis. FineSTRUCTURE R scripts based on the coancestry matrix inferred from ChromoPainter were conducted to construct the finer-scale population structure *via* heatmap, clustering dendrogram, and PCA.

### ChromoPainterv2 and GLOBETROTTER Admixture Modeling

We performed a GLOBETROTTER (Hellenthal et al., 2014) analysis for Mongolian subgroups to obtain haplotype-sharing-based evidence of admixture. Using these haplotypes from SHAPEIT2, the “chunk length” output was obtained by running ChromoPainterv2 across all chromosomes. Using the chunk length output and painting samples, we ran GLOBETROTTER to estimate admixture date by running 100 bootstrap replicates, assuming that there is detectable admixture using the “pro.ind:1” and “bootstap.date.ind:1” options.

### Signals of Recent Positive Selection

The integrated haplotype score (iHS) and XP-EHH analysis were conducted to identify recent natural signatures of positive selective sweeps in the Mongolian population using the R packaged rehh2 (Gautier et al., 2017). The SNPs used in calculated iHS and XP-EHH were filtered by minor allele frequency (--maf 0.01) and snp missing (--geno 0.05). XP-EHH requires the definition of a reference population, and we chose the southern Altaic-speaking population in Guizhou and southern Tibetan-Burman population as references to explore whether there were differences in natural selection between different geographical Altaic populations and between northern and southern populations. The SNPs with maximum negative logical p value(−log(p) > 4) of iHS and XP-EHH were regarded as candidate sites under natural selection and used as test statistics. We performed the gene annotation by 3DSNP (Lu et al., 2017) and chose genes under the natural selection of the Mongolian population to conduct Gene Ontology (GO) enrichment analysis *via* DAVID Bioinformatics Resources (Huang da et al., 2009a; Huang da et al., 2009b) and searched for related PheWAS traits and gene expression information from the global databases GeneATLAS (http://geneatlas.roslin.ed.ac.uk/) and GTEx (https://www.gtexportal.org/home/index.html), respectively.

## Results

### Population Genetic Substructure Showing the West-East Admixture Cline

**FIGURE 1:**
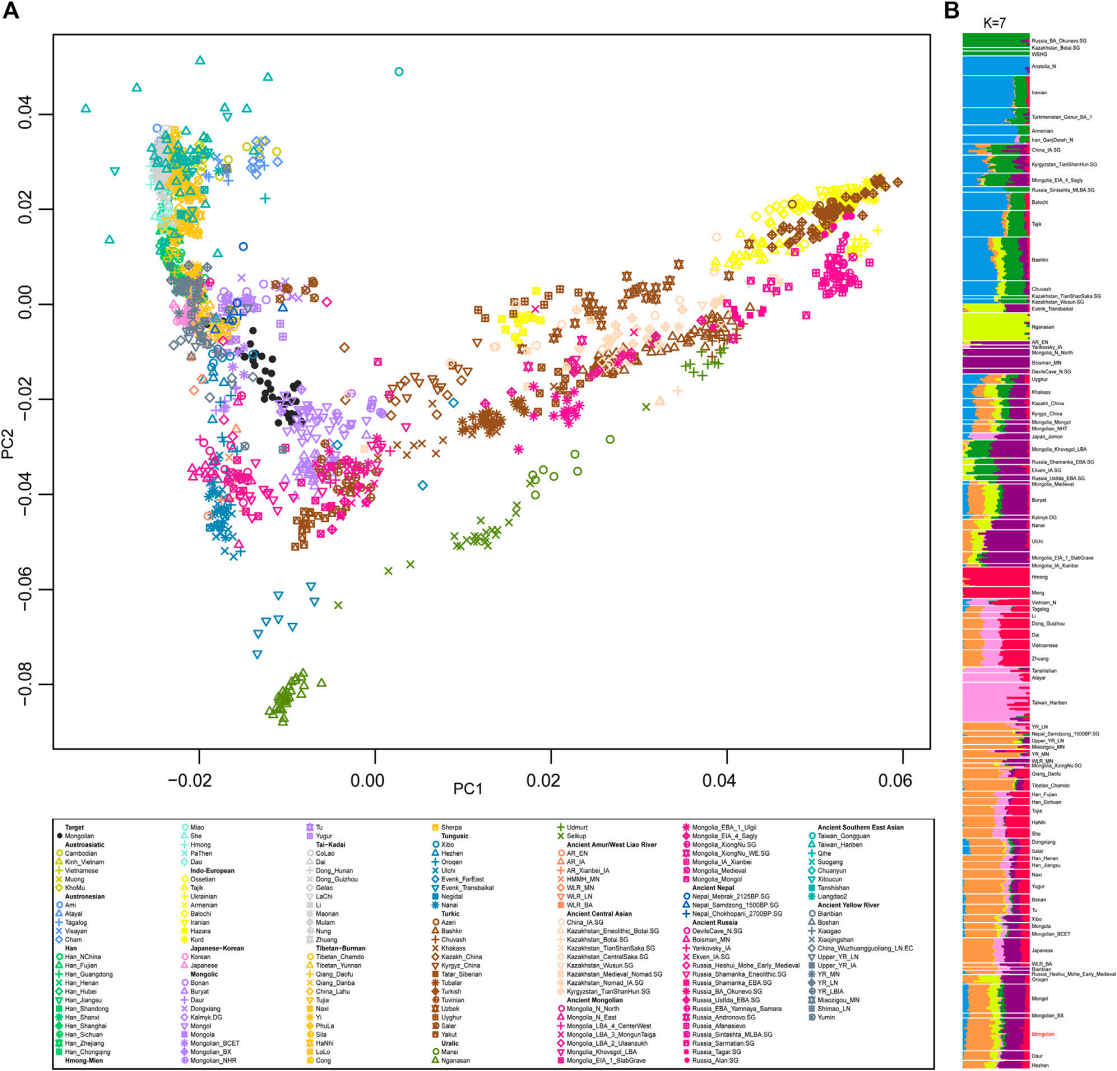
The population structure of modern and ancient populations in Eurasia based on genome-wide data. **(A)**. PCA result showed an overview population relationship between modern populations and ancient populations. **(B)**. ADMIXTURE results (the lowest CV errors K = 7): ancestral components among Mongolian and modern and ancient populations in Eurasia.

We generated and filtered 39 unrelated Mongolian individuals from Inner Mongolia Autonomous Region and merged the data with that published on modern and ancient populations in Eurasia to obtain a comprehensive population profile. In a principal component analysis (PCA) of Eurasian individuals, modern and ancient Eastern and Western Eurasian populations were separated into PC1 and PC2 split Eastern Eurasians along a north-south cline with Tungusic and Mongolic speakers who also connecting with the west-east Eurasian cline (Figure 1A). Mongolian individuals were scattered between Mongolic-speaking groups in China and ancient Mongolians, and a clear substructure was observed. To obtain a more focused Eastern Eurasian genetic profile, we removed Western Eurasian populations and the Mongolian population was stratified more obviously.

A model-based populations clustering analysis using ADMIXTURE showed a similar pattern (Figure 1B). Overall, the proportions of ancestry components associated with Eastern or Western Eurasians were well concordant with the results of PCA. The Mongolians derived most of their Eastern Eurasian ancestry from two components: one was most enriched in Sino-Tibetan speakers and the other was most represented by Mongolia_N_North that is Neolithic hunter-gatherers in Mongolia. The level of southern Eastern Eurasian-related ancestry represented by Hmong and Taiwan_Hanben in Mongolians was roughly higher than that of Mongols and Buryat. In addition, a small proportion of Western Eurasian-related ancestral component was detected in all Mongolians and Tungusic speakers. The level of admixture proportion of Western Eurasian and Eastern Eurasian in Mongolian intermediated between Mongols and previously studied Mongolians.

To obtain a more elaborate genetic structure of Mongolians, we conducted the IBD (identified by descent) analysis and pairwise *f*_*4*_ statistics of all individuals (Supplementary Figures S2–S8). Taking results from PCA, admixture, pairwise IBD, and pairwise *f*_*4*_ statistics into careful consideration, we grouped the Mongolian population into three subgroups for subsequent analysis, marked as Mongolian_inner who clustered with Mongolian speakers in China, Mongolian_mid, and Mongolian_outer clustered with Mongols and closed with Tungusic populations.

### The Differentiated Genetic Affinity and Continuity Within Mongolian Subgroups

**FIGURE 2:**
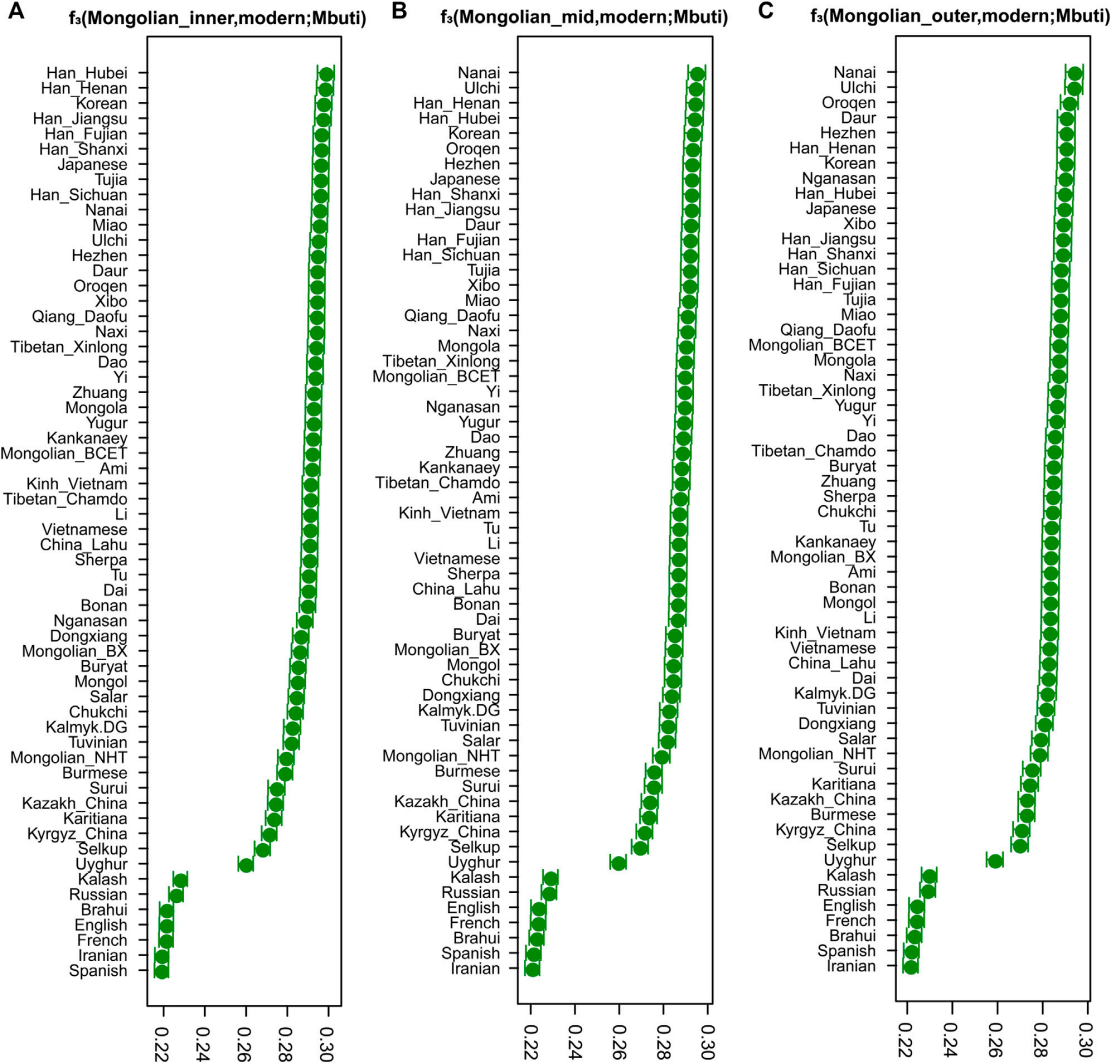
The results of three-population statistic. The shared genetic drift between modern Eurasian populations and Mongolian subgroups.

To quantitatively evaluate the genetic differences among three Mongolian subgroups and other modern and ancient Eurasian populations, we calculated the pairwise Fst genetic distances using the smartpca program (Supplementary Table S1). The genetic structure was confirmed by Neighbor-Joining Trees based on Fst (Supplementary Figure S10) results (Gautier et al., 2017), showing the different genetic affinities with other modern populations among those three Mongolian subgroups. Overall, three Mongolian subgroups showed lower genetic differences with other Mongolic-speaking groups and Tungusic populations. The Mongolian_inner was prone to cluster with Mongola_HGDP and Mongolian_BCET (Zhao et al., 2020) that belongs to Inner Mongolians and shares more genetic drift with East Asians, as shown in a previous study, and the Mongolian_outer group possessed a much closer genetic affinity to Mongols and Mongolian_BX who is Mongolian_Chahar and harbors more Western Eurasian-related ancestry than Mongolian_BCET, which was consistent with results of *f*_*4*_(*Mbuti.DG*, *X*; *Mongolian_sub*, *Mongolian_BXBC/Mongolian_HNT/Mongolian_TE*) reflecting as no significant Z (Supplementary Tables S4, S5). The Mongolian_outer showed a similar genetic profile to Mongols with a higher genetic difference with Sino-Tibetan population and southern East Asian populations and lower genetic difference with populations harboring Western Steppe-related ancestry compared to Mongolian_inner and Mongolian_mid (Supplementary Table S1). Consistent with the pattern of genetic variations that showed in PCA and Fst and the shared ancestral components observed in ADMIXTURE, the result of outgroup *f*_3_ statistics (Figure 2) in the form of *f*_*3*_ (Mongolian_sub, modern Eurasian; Mbuti) showed that Mongolian_inner possessed the most shared ancestry with modern Han groups and Mongolian_outer had strong genetic drift with Tungusic populations, while Mongolian_mid shared closer genetic affinity with Han and Tungusic populations. The genetic affinity profile also demonstrated that, in outgroup *f*_*3*_ (Mongolians, ancient Eurasian; Mbuti) (Supplementary Figure S9), Mongolian_outer shared the most significant genetic drift with ancient Northern Asian hunter-gatherers (previously called ANA or AEA), while Mongolian_inner had a closer genetic affinity with populations harbored Neolithic farmers related ancestry, suggesting an extent of long-term genetic continuity in Northeast Asia and the communication between Northeast Asian hunter-gatherers and millet farmers of North China. In addition, the shared genetic drift with three Mongolian subgroups in the Eastern Eurasian populations was stronger than that in Western Eurasians, indicating the deeper Eastern Eurasian lineage of Mongolian. The phylogenetic relationships between the studied three Mongolian subpopulations and modern Eurasian populations were further confirmed by a TreeMix-based phylogenetic tree. Among a large reference population set consisting of 47 Eurasian populations as representatives from the main language families and Mbuti as the root, we also identified a gene flow event from Tungusic into the Buryat population but not into Mongolian (Supplementary Figure S11A). When including fewer reference populations, one western gene influx flow into Mongol and the other gene flow from Western Eurasian into Eastern Eurasian was identified in the Tuvinian population of Siberia (Supplementary Figure S11B).

The genetic differentiation and affinity profile among three Mongolian subgroups was further certified by *f*_*4*_ statistics test in the form of *f*_*4*_(*Mbuti.DG*, *X*; *Mongolian_sub1*, *Mongolian_sub2*) (Supplementary Table S3), showing the significant difference in sharing affinity with ancient and modern East Asians in China between Mongolian_inner and Mongolian_outer. Mongolian_inner harbored more ancestry related to millet and rice farmers than Mongolian_mid and Mongolian_outer. The result provided evidence that Mongolian_outer harbored more Western Steppe-related ancestral components than Mongolian_inner and Mongolian_mid. Interestingly, there were differences in sharing genetic affinity to WSHG (Western Siberian Hunter-Gatherers) and Mesolithic hunter-gatherers in Japanese population (Japan_Jomon) and Iranian Neolithic farmers among these Mongolian subgroups. The additional Iranian-related ancestry was detected in ancient Mongolian populations after the Bronze Age and decreased in the modern Mongolian subgroups; notably, the level of Iranian-related ancestry in Mongolian_outer and Mongolian_mid was roughly equal to populations associated with the Late Bronze Age Ulaanzuukh (1450–1150 BCE) and Early Iron Age Slab Grave (1000–300 BCE) cultures in eastern and southern Mongolia (Supplementary Tables S6, S7D). The results of *f*_*4*_(*Mbuti.DG*, *X*; *Mongolian_sub1*, *Mongolian_sub2*) (Supplementary Table S3), *f*_*4*_(*Mbuti.DG*, *X*; *Mongolian_sub*, *Mongol/Mongola_HGDP*) (Supplementary Table S4), and *f*_*4*_(*Mbuti.DG*, *X; Mongolian_sub*, *Mongolian_BCET/Mongolian_BX/Mongolian_NHT*) (Supplementary Table S5) did provide a robust evidence of the differentiation of sharing genetic affinity with Mongols and Inner Mongolians among three Mongolian subgroups, showing a similar genetic profile with Mongola_HGDP and Mongolian_BCET of Mongolian_inner and analogical genetic structure with Mongols and Mongolian_BX of Mongolian_outer.

To further reveal the different genetic affinities of Mongolian-related populations, we used a distantly related set of outgroups. We observed a significant population stratification in three Mongolian subgroups and genetic heterogeneity in modern and ancient Mongolian-related populations except for Mongolian_inner that showed the genetic homogeneity with Inner Mongolians (Mongola_HGDP and Mongolian_BCET), Mongolian_BX that showed the genetic homogeneity with Mongolian_outer/Mongol/Mongolia_Medieval, and Buryat that showed the genetic continuity with Mongolia_Medieval (Supplementary Table S8A). We obtained a subtler population structure of Mongolian-related populations when we repeated the qpWave analysis adding outgroups that are genetically closer to the test groups. With this more powerful set of outgroups, Mongol and Buryat also provided evidence of not being pairwise clades with the remaining groups (Supplementary Table S8B), while Mongolian_BCET still displayed a close relationship with Mongola_HGDP/Mongolian_inner. Thus, beyond the broad observation of genetic affinities between three Mongolian subgroups, we also observed subtle ancestry heterogeneity in Mongolia since Bronze Age. Mongolian_inner showed continuity with Xiongnu populations in Iron Age and Mongolian_mid and Mongolian_outer showed some extent of continuity with Xiongnu, which was further confirmed in the results of *f*_*4*_(*Mbuti*, *X*; *Mongolian_inner*, *Mongolia_XiongNu.SG*) (|Z| < 3) and *f*_*4*_ (*Mbuti*, *X*; *Mongolian_mid/Mongolian_outer*, *Mongolia_XiongNu.SG*) ( part of |Z| < 3) (Supplementary Table S7). In addition, three Mongolian subgroups showed evident genetic continuity with Medieval Mongolian and the ancestry related to Han increased in modern Mongolians since the Yuan Dynasty.

**FIGURE 3:**
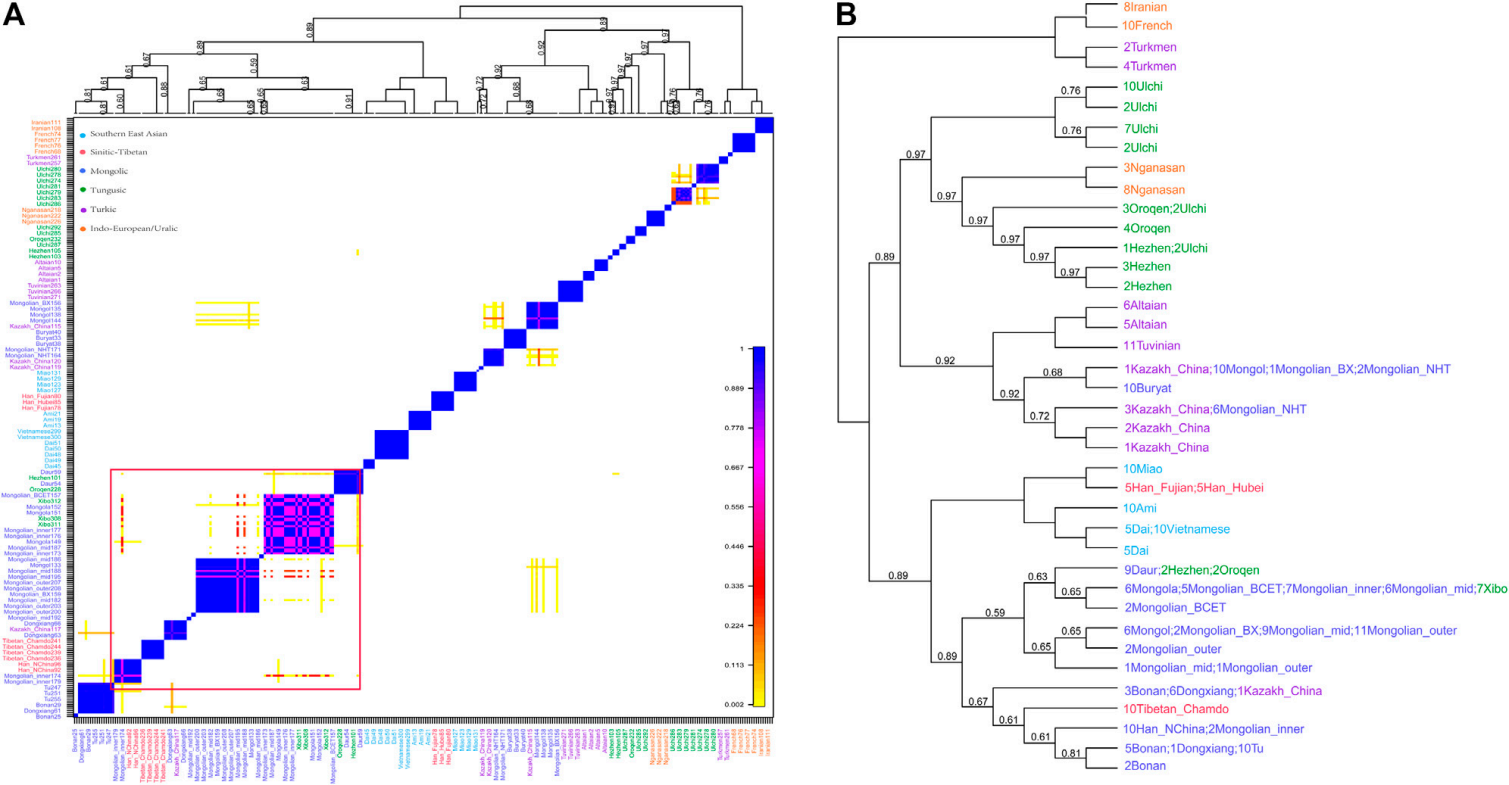
Pheatmap of sharing haplotypes and clustering dendrogram and by FineSTRUCTURE based on the chunk length.

The phased Mongolian and Eurasian populations data were also used to conduct haplotype-based fineSTRUCTURE and the finer-scale population structure of Mongolian was further comprehensively characterized. The inferred polygenetic tree showed that Mongolian_inner clustered with Mongola_HGDP and Mongolian_BCET, one part of Mongolian_mid clustered with Mongola, and the others clustered with Mongol, while Mongolian_outer was clustered with Mongolian_BX and Mongol (Figure 3B). Besides, the pattern of shared haplotypes based on the ChromoPainter showed prominent sharing haplotypes among Mongolian_outer, Mongolian_BX, and Mongol and remarkable sharing haplotypes among Mogolian_inner and Mongola (Figure 3A). PCA calculated from the coancestry matrix generated by fineSTRUCTURE also confirmed the west-east cline of Eurasians and the north-south cline of Eastern Eurasians (Supplementary Figure S13).

### The Admixture History of the Mongolian Population Based on Allele Frequency and Haplotype-Based GLOBETROTTER

**FIGURE 4:**
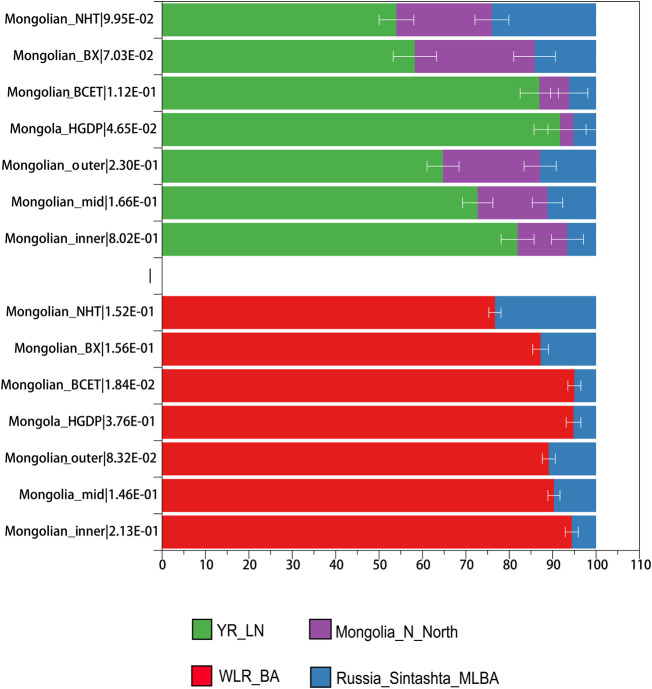
qpAdm-based admixture models for Mongolian subgroups.

We performed allele frequency-based three-population (*f*_*3*_) tests to characterize the admixed gene pools of three Mongolian subgroups. Testing all possible pairs of 115 present-day “source” groups and 117 ancient “source” groups, we detected highly significantly negative *f*_*3*_ statistics (*f*_*3*_ ≤ −3 standard error; Supplementary Table S2), providing unambiguous evidence that the target population is a mixture of groups related, perhaps deeply, to the source populations. Reference pairs with the most negative *f*_*3*_ statistics, for the most part, involved one Eastern and one Western Eurasian group (including Neolithic Iranian farmers and Chalcolithic Iranians to represent West/South Asian-related ancestry), supporting the qualitative impression of east-west admixture from PCA and ADMIXTURE analyses. To highlight the difference among Mongolian subgroups, we looked into *f*_*3*_-results with representative reference pairs comprising ancient Eurasians (Sintashta to represent the steppe Middle and Late Bronze Age ancestry and Chalcolithic Iranians to represent South Asian-related ancestry, Ulchi and Han, and ancient Mongolia to represent Eastern Eurasian-related ancestry). Farmer-related ancestry was the best representation of Eastern Eurasian ancestry for Mongolian_inner compared to Ulchi; farmer-related and Neolithic hunter-gatherers–related ancestry (Ulchi is regarded as the most genetic homogeneous population with Neolithic hunter-gatherers of DevilsCave) both represented ancestries related to Eastern Eurasian well in Mongolian_mid and Mongolian_outer. Considering the admixture events and sources that we observed in Mongolian subgroups, we applied qpWave/qpAdm to validate different proposed admixture scenarios and ancestral proportions. In the two-way mixture model of Western Steppe populations and Eastern Eurasians (Figure 4, Supplementary Table S9A), Russian_Sitashta_MLBA and WLR_BA, a mixture of Neolithic hunter-gatherers and millet farmers, approximated the Mongolian populations well (*χ*2 *p* ≥ 0.05), while the model of Eastern Eurasian simply represented by Neolithic hunter-gatherers (Mongolia_N_North and DevilsCave_N, AR_EN) or millet farmers (YR_LN) and farmers in West Liao River (WLR_MN) mostly failed, indicating that Neolithic hunter-gatherers, millet farmers, and Western Steppe populations contributed to the formation of Mongolian population together and the gene flow from the population related to millet farmers into the gene pool of Mongolian continued to today. The ancestral proportion of Western Steppe in those Mongolian subgroups was distinct, showing the parallel genetic makeup of Mongolian_outer and Mongolian_BX harboring a higher level of Western Steppe ancestry (10.9%, 12.8% Russian_Sitashata_MLBA/11.6%, 11.5% Mongolia_EBA_2_Chemurchek, a mixture population with Western Steppe), and the proportion of the ancestry in Mongolian_inner, Mongola_HGDP, and Mongolian_BCET were similar (5.6%, 5.2%, and 5% Russian_Sitashata_MLBA, respectively), the proportion in Mongolian_mid intermediated between Mongolian_inner and Mongolian_outer, coinciding with the population structure mentioned above. A more complex three-way model of YR_LN + Mongolia_N_North + Russia_Sintashta_MLBA fitted all Mongolian groups (χ2 *p* ≥ 0.05) (Supplementary Table S9B) but showed prominently various proportions of YR_LN and Mongolia_N in Mongolian subgroups, which also shown in two admixture models of millet farmers (YR_LN) + Russian_Sitashta_MLBA (χ2 P (Mongolian_inner/Mongola_HGDP/Mongolian_BCET) > 0.01), reflecting minor heterogeneity in the Eastern Eurasian source of Mongolians. Considering that we observed a gene flow signal from Iranian-related populations, all subpopulations were fitted by three models with YR_LN + Mongolia_Khovsgol_LB + Turkmenistan_Gonur_BA_1 (3.8–6%) when we added the third ancestral source of Turkmenistan_Gonur_BA_1 where is the key EBA site of the Bactria-Margiana Archaeological Complex (BMAC) culture. The legacy of the spread during the Early Iron Age was mediated by increased contact and mixture with agropastoralist populations in the region of Turan and then introduced into northwestern Mongolia along the Inner Asian Mountain Corridor. Overall, several ancestral sources contributed to the formation of modern Mongolian and the population structure was the result of different proportions of ancestries.

We reconstructed the deep demographic history using qpGraph. Mbuti, Denisovan, Onge and Tianyuan were included to explore the basal model; Early Bronze Age Afanasievo and Chemurchek, Neolithic hunter-gatherers in Mongolia Plateau, millet farmers (YR_LN), Tibetan Plateau, and Iron Age Hanben were used as ancestral source proxies from Western Eurasian, Mongolia, millet farmers in Yellow River, and southern populations. We found that Mongolian subgroups could be modeled as the mixture of EBA_Chemurchek (34–37%) derived from Western Steppe herders (47–55%) and Mongolia’s Neolithic hunter-gatherers related ancestry and Han-related ancestry (63–66%) (Supplementary Figure S12). Our qpGraph models were compatible with qpAdm results and further supported the fact that Western Eurasian herders, ANA, and millet farmers contributed to the genetic formation of modern Mongolians.

The ALDER method based on weighted linkage disequilibrium statistics also provided evidence of population structure within Mongolians (Supplementary Table S10). ALDER demonstrated multiple admixture sources from southern populations, Han, Tungusic speakers, and populations harboring Western Eurasian-related ancestry. Overall, the admixture events of Eastern and Western Eurasians occurred in a historic period (∼400–∼1700 years ago), which were consistent with the extensive western-eastern communication along the Silk Road (Yao et al., 2004; Liu et al., 2021) and the western expansion of the Mongol empire. ALDER detected extra admixture events between Tungusic/Turkic/Indo-European speakers and southern populations around ∼170–∼1700 years ago. Intriguingly, the admixture signal from Han was just detected in Mongolian_outer with admixture time ranging from ∼600 to ∼1000 years ago, inferring that the recent Han-related ancestry flowed into the Mongolians during the Late Tang Dynasty to the Yuan Dynasty when the Khitans controlled large areas of the Eastern Steppe and the Khitan empire fell to the Jurchen’s Jin Dynasty, which was then conquered in turn by the Mongols in 1234 CE. Companied by the expedition to the West by Mongol nobles, the flow of people groups was more frequent than ever before in Eurasia in the 13th century.

We further performed haplotype-based GLOBETROTTER to obtain a high-resolution characterization of the admixture landscaped of three Mongolian subgroups. All targets showed robust signals of west-east admixture (Supplementary Table S11). The west-east admixture event in subgroups could be traced back to 29–40 generations, with the inferred majority contributing Eastern Eurasian sources ranging from 77 to 87%. Mongolian_inner derived Eastern Eurasian ancestry from Han-related ancestry, while Mongolian_mid and Mongolian_outer retained Eastern Eurasian ancestry from Northeast Asia. The different Eastern Eurasian ancestral surrogates in Mongolian subgroups were in line with admixture models of qpAdm/ALDER. Meanwhile, GLOBETROTTER identified the second less strongly signaled north-south admixture event.

### The Paternal/Maternal Lineages of Mongolian

We assigned 39 mitochondrial genomes based on 4,198 maternal lineage-informative SNPs and 33 Y-chromosomal genomes based on 22,512 paternal lineage-informative SNPs (Supplementary Table S12). The maternal mtDNA lineages of Mongolians were diverse, with lineages significantly enriched in present-day East Asian populations (A, B4, C4, D4, F1, G, M, and N), showing terminal lineage frequencies ranging from 0.0256 to 0.0513 (G2a5: 2); B4, C4, D4, and F1 were prevalent in the Mongolian population. From the paternal perspective, 24 different terminal paternal lineages with frequencies ranging from 0.0303 to 0.1212 (C2b1a3b∼: 4). Siberian-dominant paternal lineage was detected (C2b1a and C2c1a). In addition, more East Asian Y-chromosomal founding lineages were identified in Mongolians with dominant lineage O2a2b1a2. To further validate the potential sex bias admixture in the Mongolian population, we used qpAdm to estimate the sex bias Z-score. We observed positive Z sex bias scores in different two-way admixture models focused on Mongolians, which suggested a male-dominated admixture of Han-related ancestry.

### The Natural Selection Signal and Functional Genes in Mongolian

**FIGURE 5:**
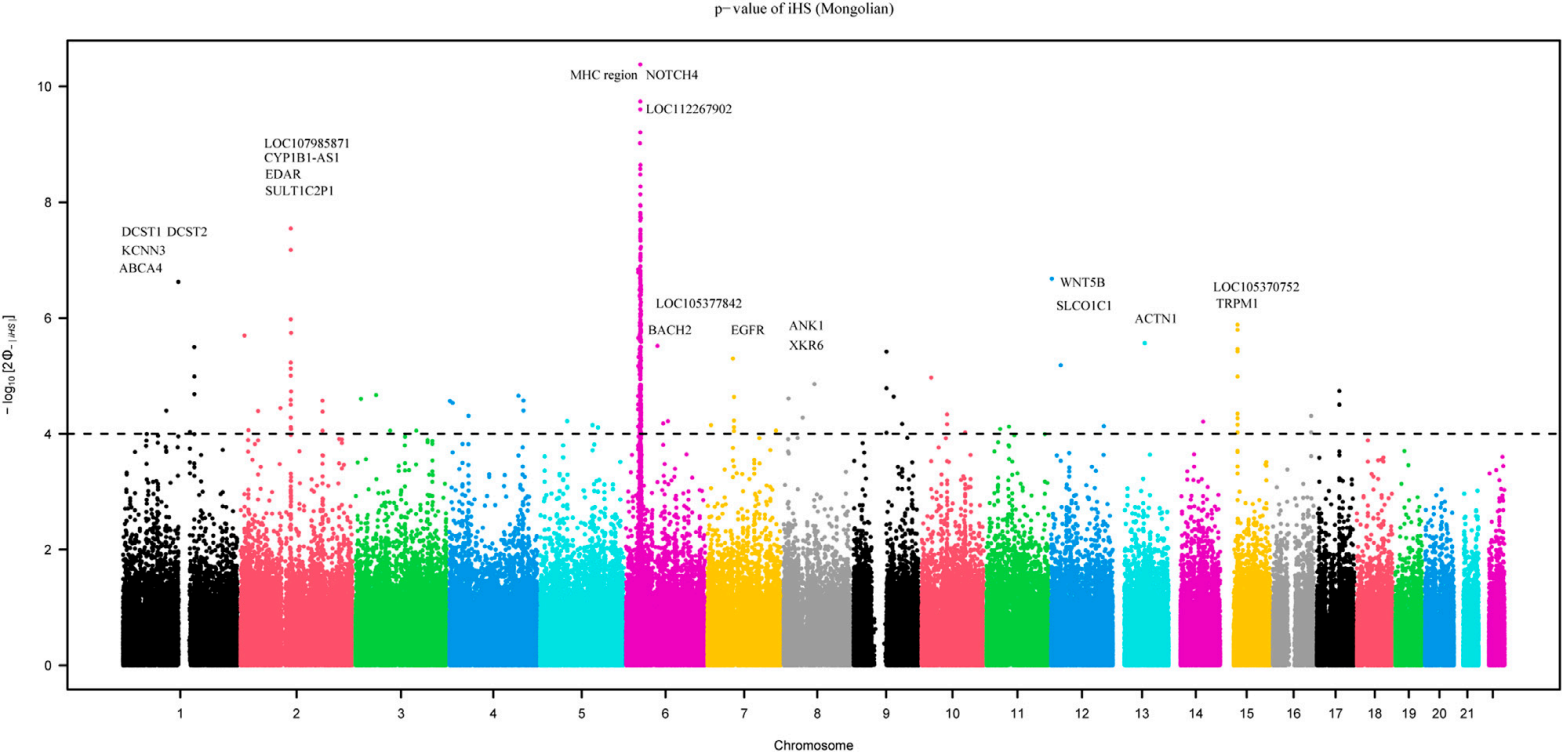
The result of recent natural signatures of positive selective sweeps in Mongolian population based on iHS showed the strongest positive selection region in MHC region.

**FIGURE 6:**
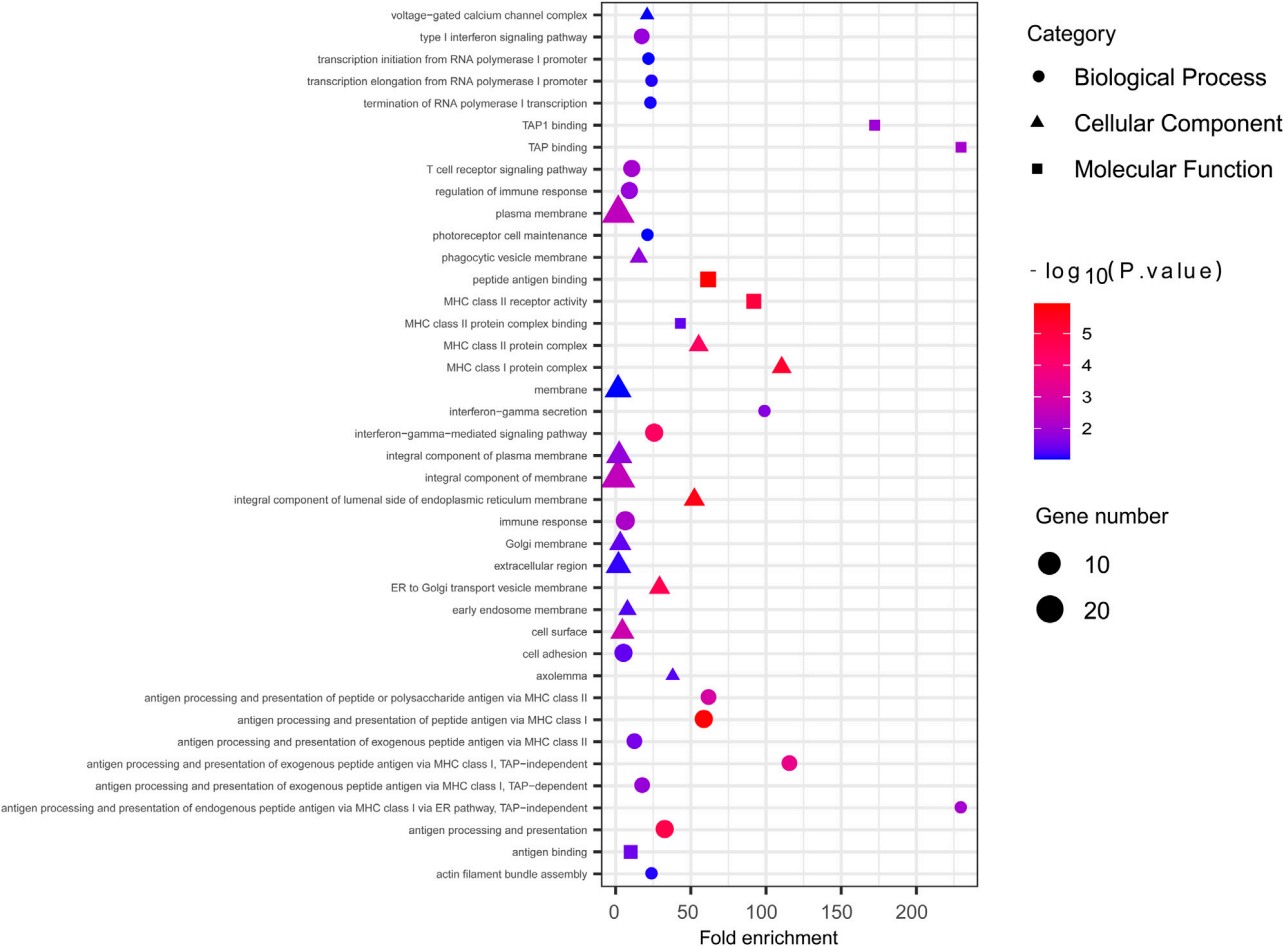
The GO enrichment analysis of Mongolian showed genes with significant natural signal were mostly enriched in membrane-associated cellular component.

We employed the iHS test to identify recent natural signatures of positive selective sweeps in the Mongolian population. Some differences of loci under natural selection detected by iHS among Mongolian subgroups existed, the GO enrichment of Mongolian subgroups’ genes with significant natural selection, however, all showed mainly enriched in cellular component with the membrane (Supplementary Table S13B–D; Supplementary Figure S14). Therefore, considering the small sample size of subgroups that is likely to cause the deviation of detected selection signals and the homogenous Mongolian relative to other populations, we performed the natural selection related analysis on the whole Mongolian group subsequently (Figure 5, Supplementary Table S13A). We observed the highest −log_10_p (iHS) score in the Major Histocompatibility Complex (MHC) region, indicating that genes in this region might experience strong positive selection, which has been already found in previous studies. In addition, in the gene *TRPM1* located in chromosome 15, more than 30 SNPs showed strong selection signatures (−log_10_p > 4), which indicated significant enrichment of selection in this genomic region. The *EDAR* gene (rs922452) was identified with higher |iHS|, which has shown the strong signatures of positive selection in East Asians (Kamberov et al., 2013). Notably, the alcohol dehydrogenase (*ADH*) gene cluster was not identified. The derived allele frequencies of the *ADH* gene family in those Mongolian subgroups, however, were higher and associated with the genetic affinity to Han (adjusted *R* ^2^ > 0.5, adjusted p < 0.01) (Supplementary Table S14). In addition to iHS, XP-EHH was also used to indicate the effect of local positive selection. The results of XP-EHH (southern Altaic/southern Tibetan-Burman vs. Mongolian) (Supplementary Table S13E–F) showed overlapping positive selection signals in northern Mongolian population relatively to southern Altaic populations (Mongolian_Guizhou and Manchu_Guizhou) and southern Tibetan-Burman population, including *SLC28A3*, *SLC47A1*, *LOC100506499*, *ZFPM2*, *AGBL4*, and *MHC* regions. However, there still were differences in positive selection between Mongolian relative to southern Altaic populations and Mongolian relative to southern Tibetan-Burman population. The number of loci that experienced positive selective sweeps in Mongolian relative to southern Altaic was less than that in Mongolian relative to southern Tibetan-Burman population, indicating a diverse local selection and adaption in regions. Genes subjected to natural selection were concentrated in a membrane-associated cellular component, while genes enriched in molecular function and biological processes were associated with immune response (Figure 6). Furthermore, the related traits from the GeneATLAS dataset in chromosome 6 showed immune-related traits. Gene expression of those genes was mostly focused on such immune tissue as brain, reproductive organ, skin, stomach, and spleen (Supplementary Table S15).

Animal husbandry is the main means of livelihood of Eastern Steppe herders; therefore, dairy livestock is a staple food and traditional diet style. We found that despite a pastoralist lifestyle started in Late Bronze Age, the Mongolians did not have a higher frequency of derived mutations associated with lactase persistence (*LCT/MCM6*, frequency < 0.07143), which showed a strong positive correlation with the genetic affinity to Western Eurasian (adjusted *R* ^2^ > 0.7, adjusted p < 0.05) (Supplementary Table S14). Given the dairy habit of Mongolians, we observed the derived allele frequencies of the FADS1 gene (Supplementary Table S14) intermediated between northern Han and southern Han when fatty acid desaturase (*FADS*) gene family which plays vital role in the biosynthesis of polyunsaturated fatty (Schaeffer et al., 2006; Nakayama et al., 2010; Song et al., 2013; Wu et al., 2017) has been taken into account. Due to the absence of a phenotype dataset, we could not further analyze the association of *FADS* with the high-fat dairy consumption of Mongolians.

## Discussion

We provided newly generated genome-wide SNP data of the Mongolian population from the Inner Mongolia Autonomous Region and performed a comprehensive population genetic analysis to investigate the genetic origin and admixture history. Findings from IBD segments among pairwise individuals, approximate ancestral composition differences from ADMIXTURE result, and pairwise *f*_*4*_(studied individual1, studied individual2; Atayal/Han_NChina/Tibetan_Chamdo/Ulchi/Mongol/Mongola, Mbuti) suggested that our focused Mongolian existing population stratification was genetically separated into three subgroups. Overall, even though three Mongolian subgroups had a closer genetic relationship with Tungusic populations, which might result from Altaic-speaking populations—the common ancestor of Tungusic and Mongolian provided by linguistic information, there were differences in sharing genetic affinity with Eurasian populations among Mongolian subgroups. The grouped Mongolian subpopulations showed significant distinction of genetic affinity with previously studied Mongolians of Inner Mongolia Autonomous Region and Mongols. That was, Mongolian_inner had a similar genetic profile with Mongola_HGDP and Mongolian_BCET showing the most shared ancestry with modern Han groups, while Mongolian_outer genetically closed to Mongols showed the higher genetic difference with Sino-Tibetan and southern East Asian populations and lower genetic difference with populations harboring Western Steppe pastoralists related ancestry than Mongolian_inner and Mongolian_mid, and the genetic profile of Mongolian_mid intermediated between Mongolian_inner and Mongolian_outer. The *f*_*4*_(*Mbuti*, *X*; *Mongolian_sub1*, *Mongolian_sub2*), *f*_*4*_(Mbuti, X; Mongolian_sub, Mongol/Mongola_HGDP), and *f*_*4*_(Mbuti, X; Mongolian_sub, Mongolian_BCET/Mongolian_BX/Mongoliann_NHT), qpWave homogenous test did further provide evidence of the genetic structure and the diverse sharing genetic affinity to modern/ancient Mongolians among three Mongolian subgroups.

Paleogenomic studies demonstrated that the disparate genetic profile of ancient Mongolian existed at different times and geographic regions and multiple ancestral sources flowed into Mongolia Plateau shaped the higher genetic heterogeneity of ancient Mongolian: the local ANA ancestry, the ephemeral ANE ancestry, the eastward movement of Western Steppe herders in a different period, limited gene flow of Iranian-related ancestry, and recent Han-related ancestry. The intercontinental expansion of Mongols established the genetic structure that characterized the present-day Mongolic-speaking population in North Asia. Model-based populations clustering analysis of ADMIXTURE and admixture *f*_*3*_ tentatively suggested that the differentiated genetic profile of Mongolians might be the results of various ancestral sources and proportions: the Eastern Eurasian including Neolithic hunter-gatherers related ancestry (ANA, represented by DevilsCave_N/Mongolia_N_North), millet farmers related ancestry (represented by YR_LN), and relative low proportion of ancestry related to Western Steppe herders contributed to the gene pool of modern Mongolian, in agreement with previous studies (Zhao et al., 2020). The gene flow from Western Eurasian was preliminarily detected in Mongol population of TreeMix-based phylogenetic tree; the ancestral source was finally identified in qpAdm, ranging from 5.6 to 11.6% in those Mongolian subgroups; ALDER and GLOBETROTTER supported that the west-east admixture event was recently estimated in the period ranging from Tang Dynasty to Yuan Dynasty. One important point is that the truth admixture scenarios might be continuous, complicated admixture and estimated admixture only provide simply a single event, and the recent date should be paid attention. The admixture between Western Steppe pastoralists and ancient Eastern Eurasians in the Mongolia Plateau has been attested in paleogenomics studies, including Early Bronze Age Yamnaya and Afanasiveo populations showing primary culture influence and limited genetic impact and Middle and Late Bronze Age Andronovo and Sintashta with visible genetic contribution to Eastern Steppe populations and historic nomadic pastoral. What is more, the Silk Road, connecting the Eurasian continent, promoted not only prosperous western-eastern population communication and culture exchange but also genetic material flow. The rise of the nomadic empire in the historic period facilitated the population interaction of western-eastern Eurasian and farmers-pastoralists.

Neolithic hunter-gatherers and millet farmers in East Asia made a large genetic contribution to the formation of Mongolian matched by the two-way admixture model of WLR_BA that is a mixed population of Neolithic hunter-gatherers and millet farmers and Western Steppe herders or adequately modeled as YR_LN + Mongolia_N_North/AR_EN + Russia_Sintashta_MLBA or YR_LN + Russia_Sintashta_MLBA + Mongolia_N_North + Turkmenistan_Gonur_BA_1. The proportion of Neolithic hunter-gatherers contributing to Mongolian subgroups increased with the genetic affinity with Mongols; in contrast, the ancestry of Neolithic farmers dedicated to Mongolian subgroups increased with the genetic affinity with Han. The derived Eastern Eurasian ancestry (ANA) from a gene pool was similar to contemporary Tungusic speakers from Amur River Basin, suggesting a genetic connection among the speakers of languages belonging to the Altaic macrofamily (Turkic, Mongolic, and Tungusic language families) (Yunusbayev et al., 2015; Pugach et al., 2016; Chen et al., 2021; Zhang et al., 2021). The genetic connection of Mongolic and Tungusic populations was also shown in a similar pattern of the paternal Y chromosomes (Huang et al., 2018a; Huang et al., 2018b; Wen et al., 2019; Wei et al., 2018a; Yan et al., 2015; Wei et al., 2018b). Trans-Eurasian language origin hypothesis asserted that the language subfamily of Mongolic, Tungusic, Turkic, and Japonic-Korean originated from Neolithic Hongshan culture in West Liao River Basin; the Hongshan farmers in West Liao River Basin migrated westward to the Mongolia Plateau and gradually developed into nomadic style, leading to the separation of Proto-Turkic and Proto-Mongolic-Tungusic languages. However, our findings did not observe the Hongshan related ancestry in Mongolic speakers and supported the Trans-Eurasian agricultural origin and diffusion hypothesis (the two-way admixture of WLR_MN + Russia_Sintashta_MLBA failed, Supplementary Table S9A). Considering the genetic similarity continuity in ancient Northeast Asian, our established genetic landscape in Mongolians supported the potential Northeast Asian origin of the Altaic language. What's more, the genetic contribution of Han-related ancestry might be mediated by the gene flow into ancient populations in Mongolia started in the Xiongnu Regime of the Early Iron Age (Jeong et al., 2020; Wang et al., 2021). The unique geographic position of the Inner Mongolia Autonomous Region has always been the boundary between the agriculture of the Han population and the pastoral husbandry of herders. Therefore, the recorded communication between the populations related to Han and Eastern Steppe pastoralists started in Han Dynasty when the rise of the Xiongnu Regime often invaded the boundary of the Han Dynasty, which facilitated the cultural and genetic exchanges. Since the confrontation between the Han and the nomads opened up the historical situation, this kind of exchange between the agricultural people and the nomads has continued until Genghis Khan’s cavalries swept across the whole Eastern Eurasia and the exchanges between the agricultural people and the nomads reached the peak; our ALDER results also suggested gene flow from Han into Mongolian during the rise of the Mongol empire. The Han-related ancestry increased with the time transection. Sex-biased patterns of genetic admixture could be informative about gendered aspects of migration, social kinship, and family structure. We observed a clear signal of male-biased Han admixture in the Mongolian population, corresponding to the Y chromosome lineage O2a in some Mongolian individuals.

The additional ancestral source related to populations of Central Asia (Caucasus/Iranian Plateau/Transoxiana regions) flowed into Eastern Eurasian initiated in the Early Iron Age along the Inner Asian Mountain Corridor/the Tian Shan Mountains, which is detected in the Iron Age groups such as TianShan Saka, Mongolia_Chandman_IA (Jeong et al., 2019; Jeong et al., 2020). This genetic influx continued to the Xiongnu Empire and even the Early Medieval period. The westward disseminating Turkic language influenced the group in the south-eastern side of the Tian Shan Mountains, such as Wusun and Kangju (Damgaard et al., 2018). The Xiongnu population and in a later Uyghur period, Wusun and Kangju in the Tian Shan Mountains received an Iranian-related ancestry (BMAC related or Neolithic Iranian-related). Although the Iranian-related ancestry component did not largely contribute to the gene pool of the Mongolic-speaking population, it has been detected in modern Mongolians. Our modern Mongolian populations also showed a minor genetic affinity to Iranian-related populations; the genetic affinity in Mongolian populations was inferior to that in ancient populations in Mongolia Plateau since the Late Bronze Age. The qpAdm results further provided robust evidence that the subtle genetic influx was dedicated to the gene pool of modern Mongolians.

The Eastern Steppe has served as a crossroad for human population migration and cultural exchanges: the eastward expansion of Western Steppe herders since the Bronze Age (Allentoft et al., 2015; de Barros Damgaard et al., 2018; Narasimhan et al., 2019; Ning et al., 2019; Wang et al., 2019); the WSHG (West Siberian hunter-gatherers) in Central/South Asia (Jeong et al., 2019; Narasimhan et al., 2019; Wang et al., 2021); Iran-related ancestry flowed into northern Mongolia since Early Iron Age (Jeong et al., 2020). More recent historical migrations are companied by the opening of the Silk Road and the westward expansions of Turkic and Mongolic groups. The flourishing population movement facilitated the intricate formation history of the Mongolian population. Our sample was collected from Darham Mau Mingan Union Flag of Baotou of Inner Mongolia Autonomous Region, which is located in the hinterland of the Bohai Rim and the Yellow River Economic Belt and has functioned as a conduit for human migration and cultural transfer between Mongols and China so that also be characterized as an immigrant city with flourished migrations. Prosperous economic and trade activity promotes the population exchange between China and Mongols, which is also shown in the genetic profile of three Mongolian subgroups.

The detailed population origin and admixture history provide clues to understanding natural selection and functional genes. In Mongolians, we detected the strong selection signal from the MHC region, which is a key point of the human immune response. Gene enrichment analysis also supported the most enrichment related to the human immune response in terms of cellular component and molecular function. The positive selective sweeps in this region have been already identified in Han populations (Zheng et al., 2021). However, the alcohol dehydrogenase (*ADH*) gene cluster that underwent regional selective sweeps in East Asia (Ma et al., 2005; Li et al., 2008; Li et al., 2011; Allentoft et al., 2015) was not identified, and the derived allele frequency of *ADH* genes in three Mongolian subgroups showed a strong correlation with the genetic affinity to Han, indicating the possibility of introducing genes into Mongolians. The fact that Mongolians started milk consumption in the Late Bronze Age (Jeong et al., 2018; Wilkin et al., 2020) suggested that ruminant dairy pastoralism was adopted on the Eastern Steppe by local hunter-gatherers through a process of cultural transmission and minimal genetic exchange with outside groups. Ancient populations in the Eastern Steppe of different periods have a negligibly low frequency of the derived mutation with no increase in frequency over time (Jeong et al., 2018; Jeong et al., 2020). The derived mutation in modern Mongolians was still at low frequency, even if the frequency increased with the genetic affinity to Western Eurasian in subgroups. Therefore, the ability to digest large quantities of lactose for millennia in the absence of lactase persistence is remarkable, which may be related to their reportedly unusual gut microbiome structure.

## Conclusion

We generated genome-wide data from 42 Mongolians of the Inner Mongolia Autonomous Region. We first identified a significant genetic differentiation among Mongolians, who were structured into three distinct genetic clusters harboring various Western and Eastern Eurasian ancestries. Findings based on the *f*-statistics demonstrated that Mongolian subgroups possessed different Chinese Mongolian/Mongols/Tungusic/East Asian affinities, indicating successful population migration in a frontier city. The successfully fitted four-way admixture model revealed that Eastern Eurasian ancestry included Northeast Asian Neolithic hunter-gatherers related ancestry and East Asian millet farmers related ancestry and Western Eurasian ancestry included Western Steppe herders related ancestry and small Iran-related ancestry. Furthermore, the natural selection analysis of Mongolian showed that the MHC region underwent significant positive selective sweeps and the functional *ADH* and *LCT* were not identified. This study characterized the complex population admixture history of Chinese Mongolians, which shed light on the intensified interaction and mixture history of farmers and pastoralists in the boundary between agriculture of contemporaneous imperial Han and pastoral husbandry of herders. Moreover, it revealed intricate genetic structure in a frontier industrial city. The genetical structure of populations inspired that the regional positive selection with allele frequency change might be associated with the genetic affinity. It will be extremely important to expand the set of available ancient and modern genomes across the Eastern Steppe to fully reveal the population structure and history of the Eurasian Steppe and further investigate the local natural selection of functional genes.

## Data Availability

The datasets presented in this study can be found in online repositories. The names of the repository/repositories and accession number(s) can be found below: https://zenodo.org/record/5067504, doi: 10.5281/zenodo.5067504.

## Supplementary Material

Supplementary Figure S1
**(A)**. The geographic distribution of Mongolians. **(B)**. The PCA results focused on Eastern Eurasians displayed a clearer genetic structure of Mongolians.

Supplementary Figure S2 The pairwise f4 statistics in forms of f4 (studied individual1, studied individual2; Atayal, Mbuti.DG ). Z scores with 2<|Z|<6 were labeled as “+/−,” significant Z scores(|Z| > 6) were labeled as “++/−−.”

Supplementary Figure S3 The pairwise f4 statistics in forms of f4 (studied individual1, studied individual2; Han_NChina, Mbuti.DG). Z scores with 2<|Z|<6 were labeled as “+/−,” significant Z scores (|Z| > 6) were labeled as “++/−−”.

Supplementary Figure S4 The pairwise f4 statistics in forms of f4 (studied individual1, studied individual2; Tibetan_Chamdo, Mbuti.DG). Z scores with 2<|Z|<6 were labeled as “+/−”; significant Z scores(|Z|>6) were labeled as “++/−−”.

Supplementary Figure S5 The pairwise f4 statistics in forms of f4 (studied individual1, studied individual2; Ulchi, Mbuti.DG). Z scores with 2<|Z|<6 were labeled as “+/−”; significant Z scores(|Z|>6) were labeled as “++/−−”.

Supplementary Figure S6 The pairwise f4 statistics in forms of f4 (studied individual1, studied individual2; Mongol, Mbuti.DG). Z scores with 2<|Z|<6 were labeled as “+/−”; significant Z scores(|Z| > 6) were labeled as “++/−−”.

Supplementary Figure S7 The pairwise f4 statistics in forms of f4 (studied individual1, studied individual2; Mongola, Mbuti.DG). Z scores with 2<|Z|<6 were labeled as “+/−”; significant Z scores(|Z|>6) were labeled as “++/−−”.

Supplementary Figure S8 The heatmap of pairwise IBD (identified by descent) segments among Mongolian individuals.

Supplementary Figure S9 The shared genetic drift between ancient Eurasian populations and Mongolian subgroups.

Supplementary Figure S10 The genetic distance (Fst) based on smartpca showed the genetic difference in Eastern Eurasian among Mongolian subgroups.

Supplementary Figure S11 The phylogenetic relationships between the studied Mongolian populations and modern Eurasian populations based on Treemix. **(A)**. TreeMix based on relative genetic drift showed the polygenetic relationship among global populations and four-gene flow events. One gene flow occurs between Ulchi and Mongolian speaker Buryat. **(B)**. TreeMix-based phylogenetic tree including fewer references showed one western gene influx flow into Mongol.

Supplementary Figure S12 The best-fitted qpGraph-based deep population admixture history of Mongolian subgroups.

Supplementary Figure S13 PCA patterns based on the coancestry matrix of linked SNP markers.

Supplementary Figure S14 The GO enrichment analysis of Mongolian subgroups.
