# Supplementary figures and images for: Genomic Insights Into the Genetic Structure and Natural Selection of Mongolians
Source: Front Genet. 2021 Dec 8;12:735786. doi: 10.3389/fgene.2021.735786 (PMC8693022; doi:10.3389/fgene.2021.735786)

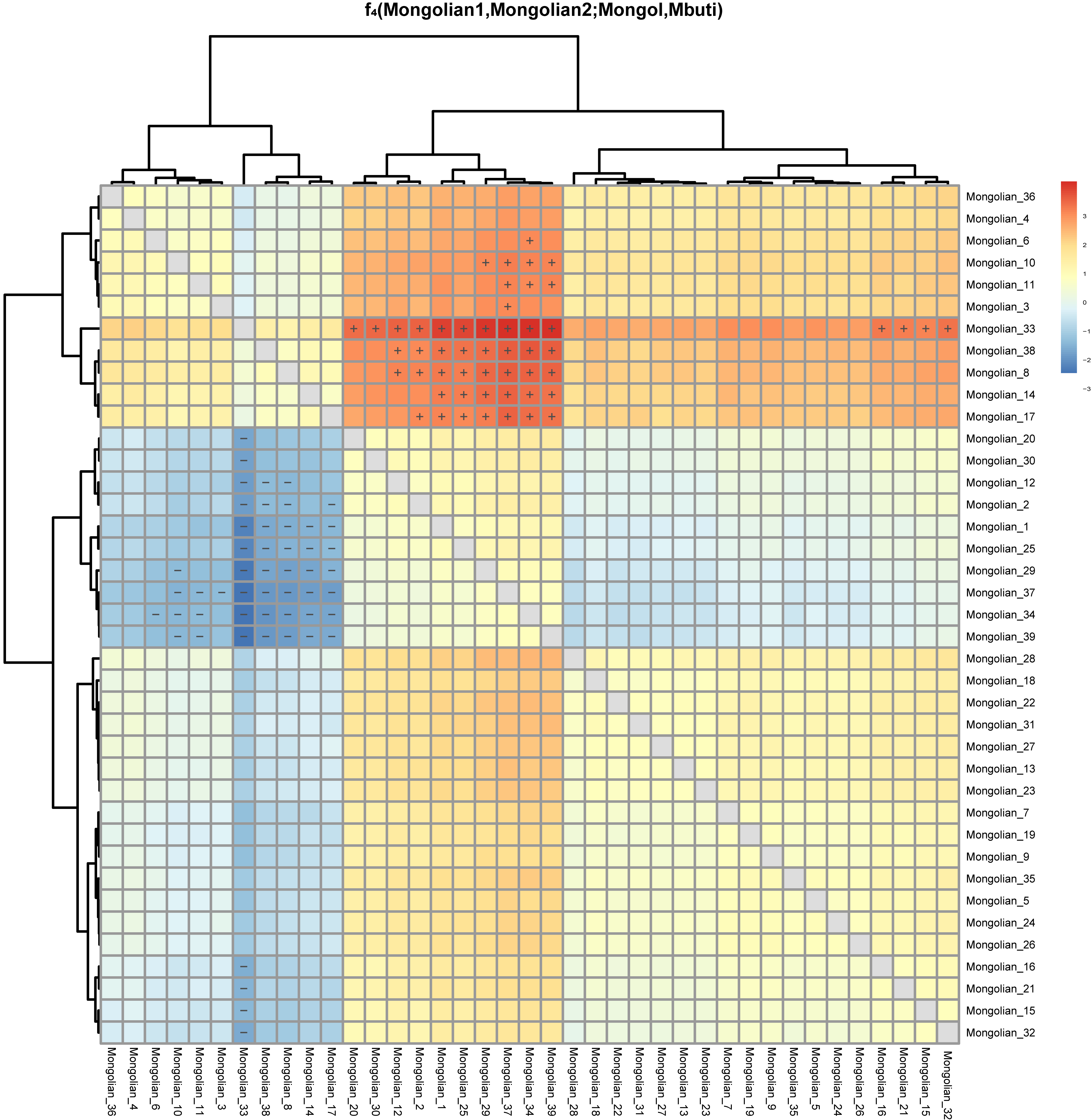

Supplement: Supplementary file 4 [file Image6.tif]

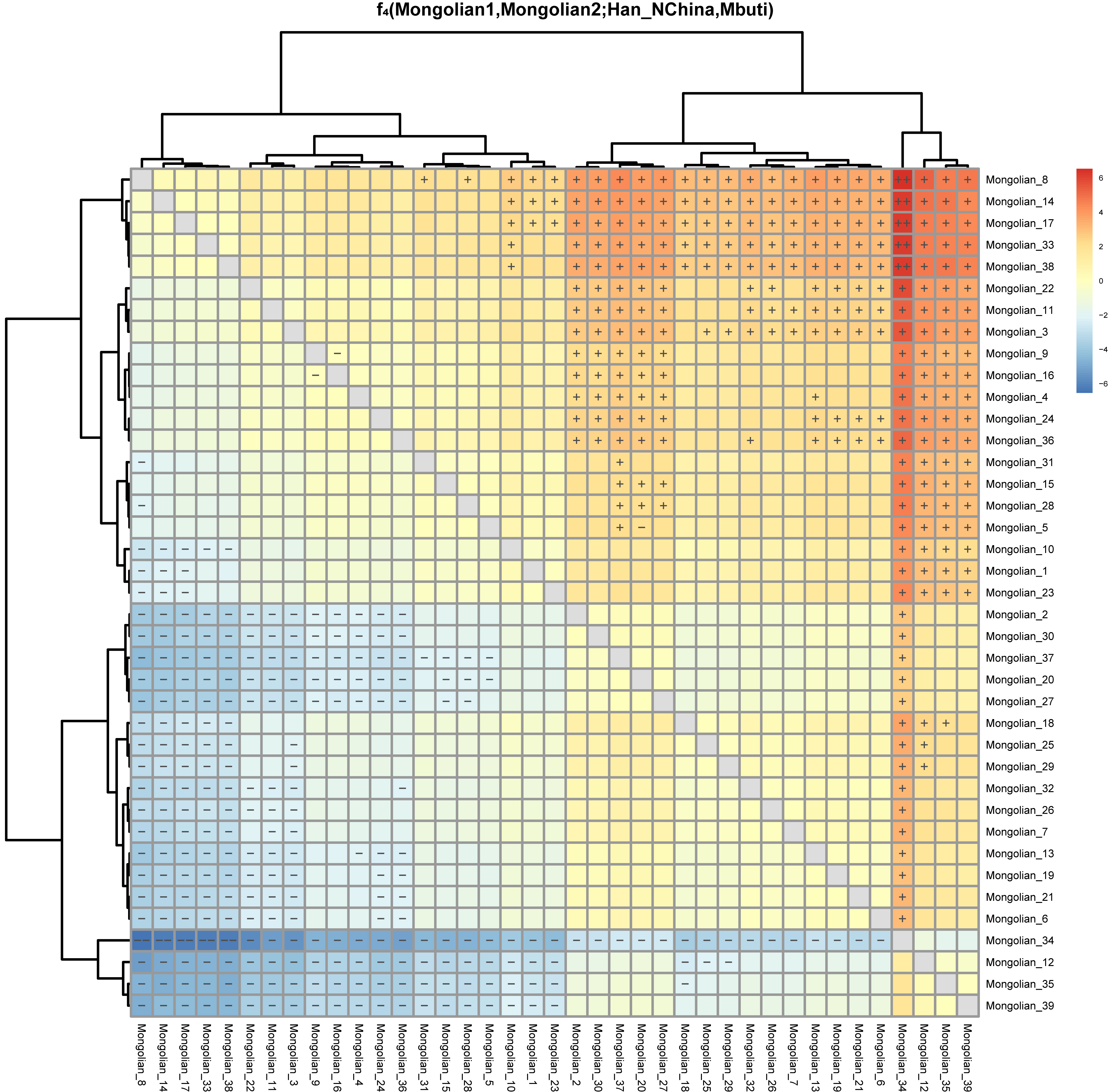

Supplement: Supplementary file 7 [file Image3.tif]

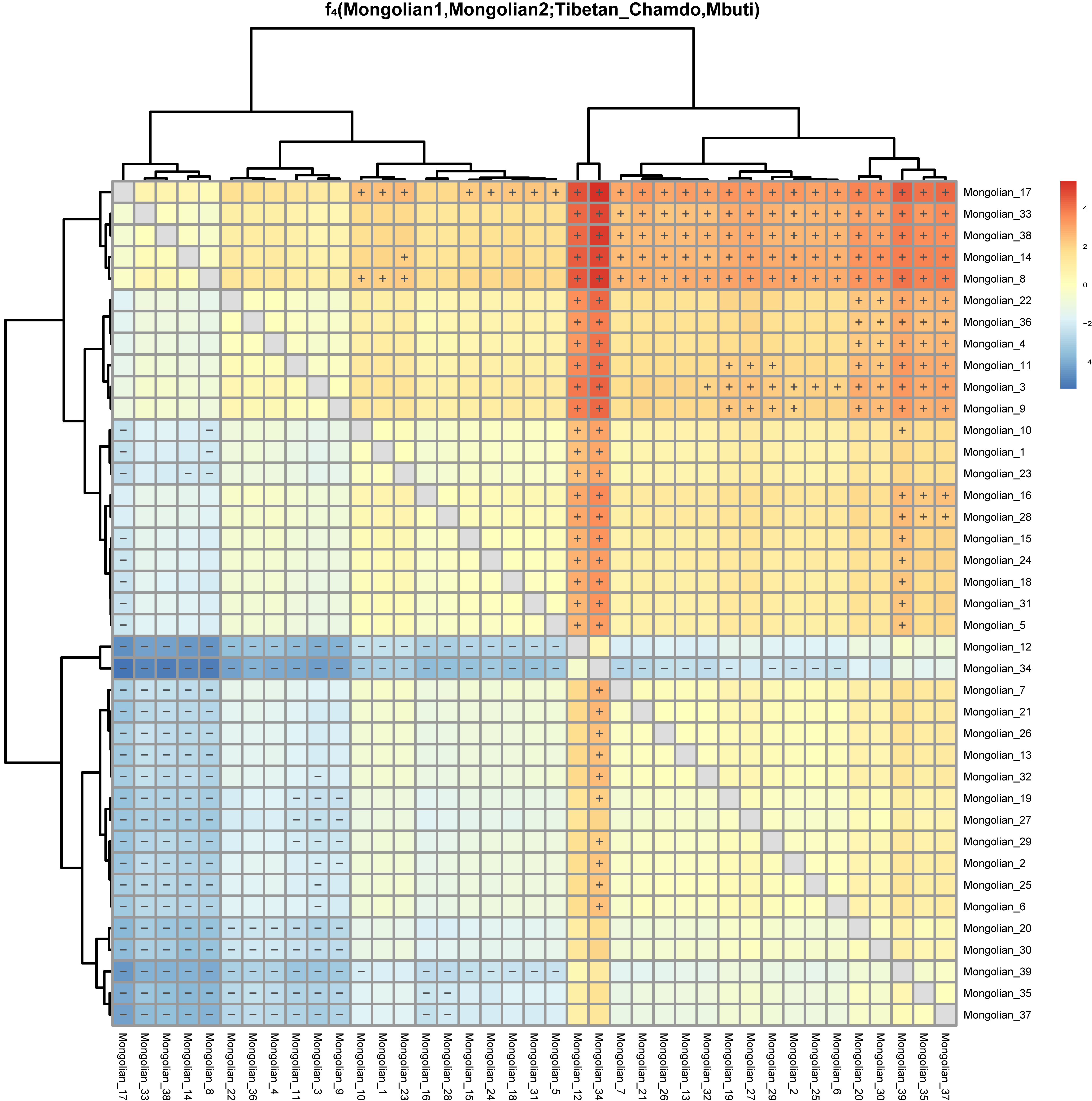

Supplement: Supplementary file 8 [file Image4.tif]

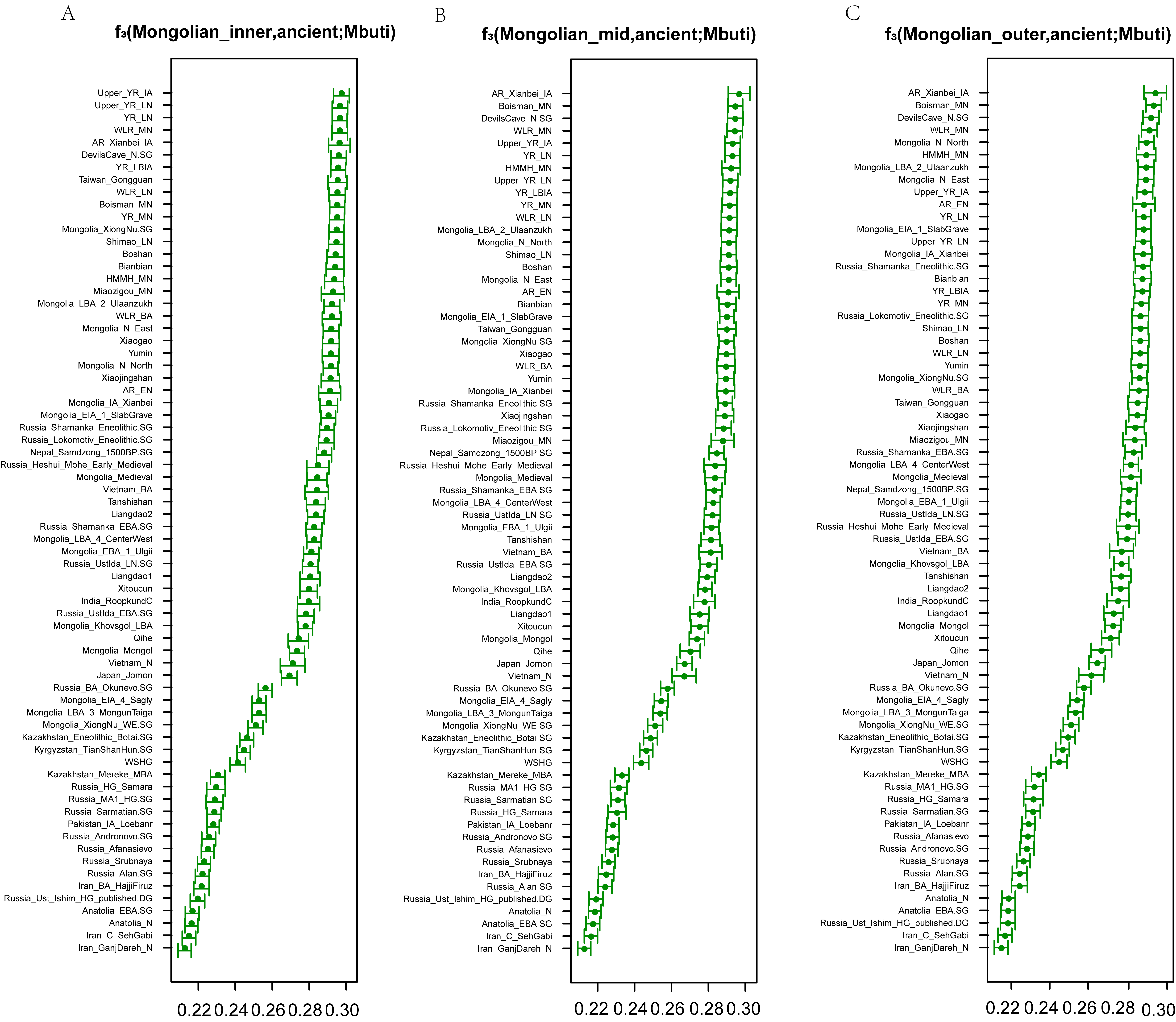

Supplement: Supplementary file 9 [file Image9.tif]

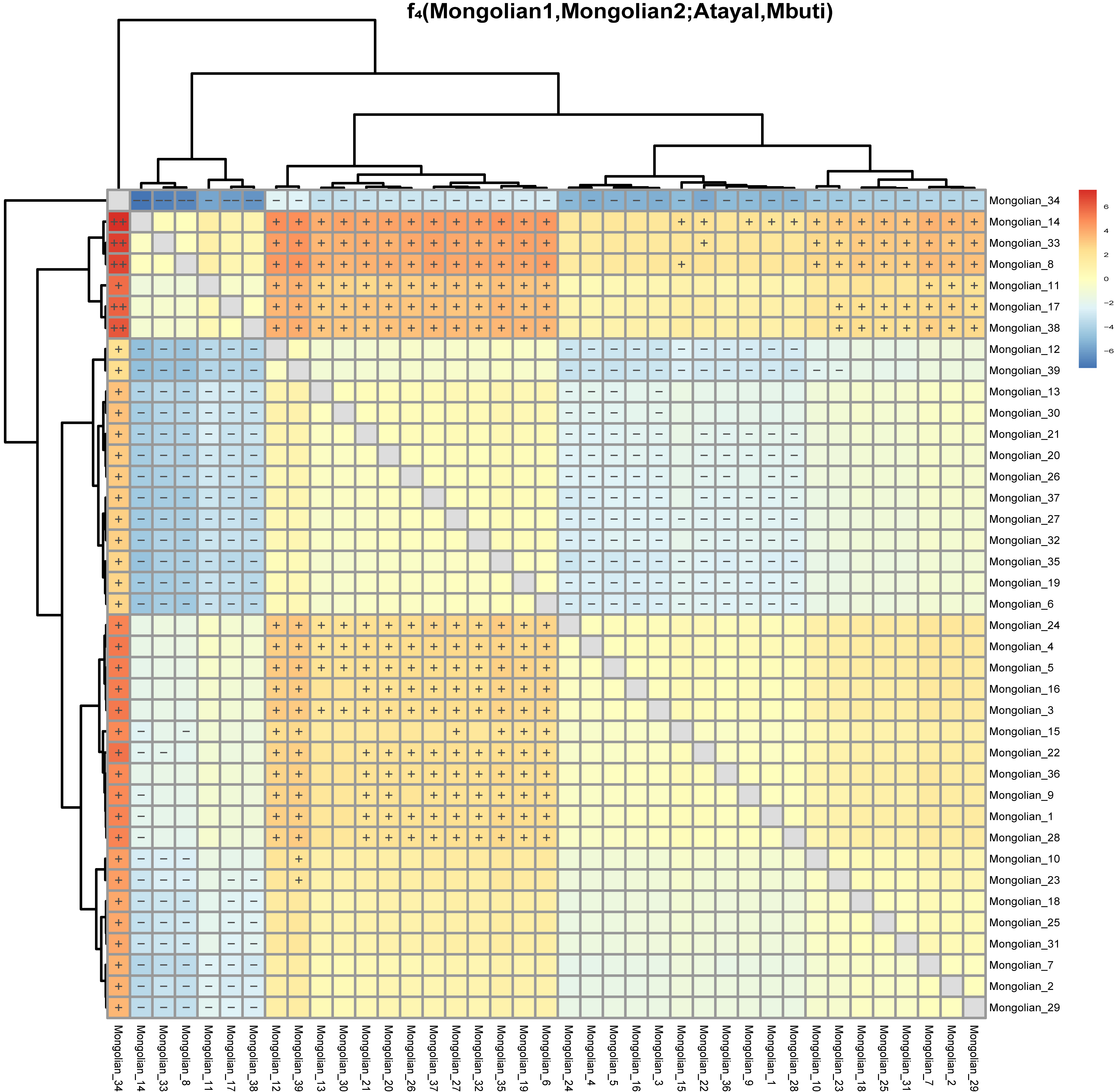

Supplement: Supplementary file 10 [file Image2.tif]

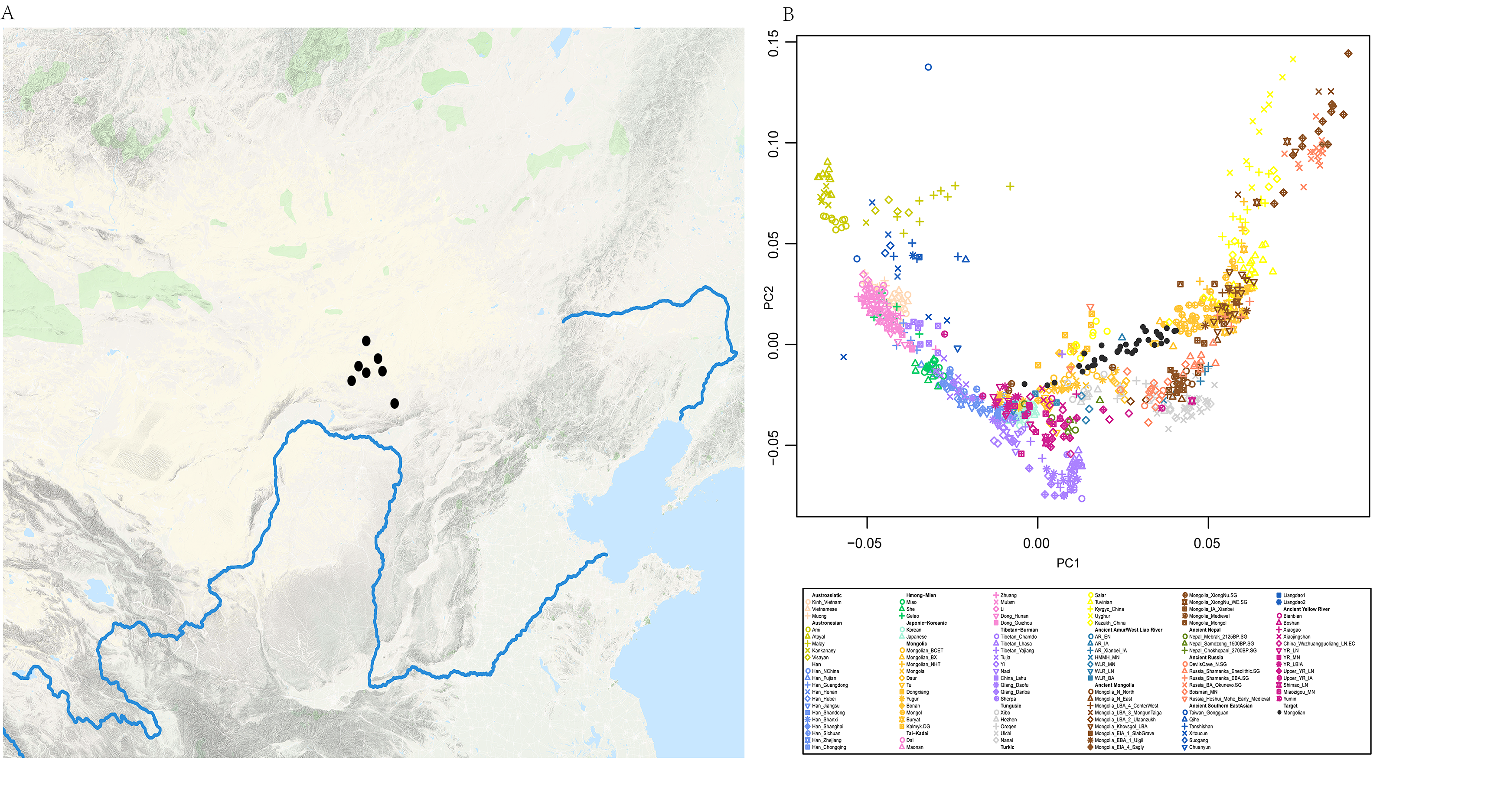

Supplement: Supplementary file 14 [file Image1.tif]

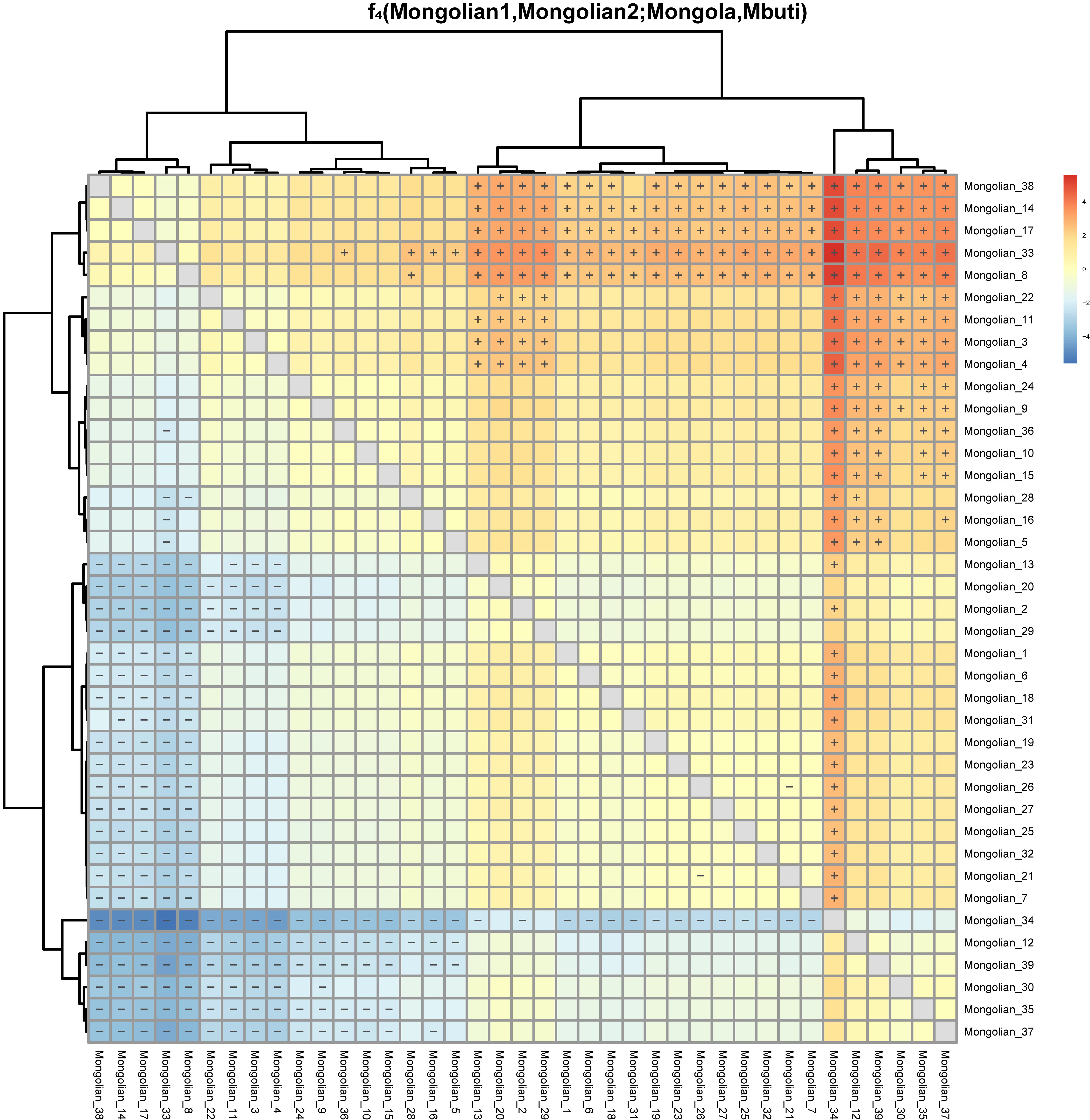

Supplement: Supplementary file 16 [file Image7.tif]

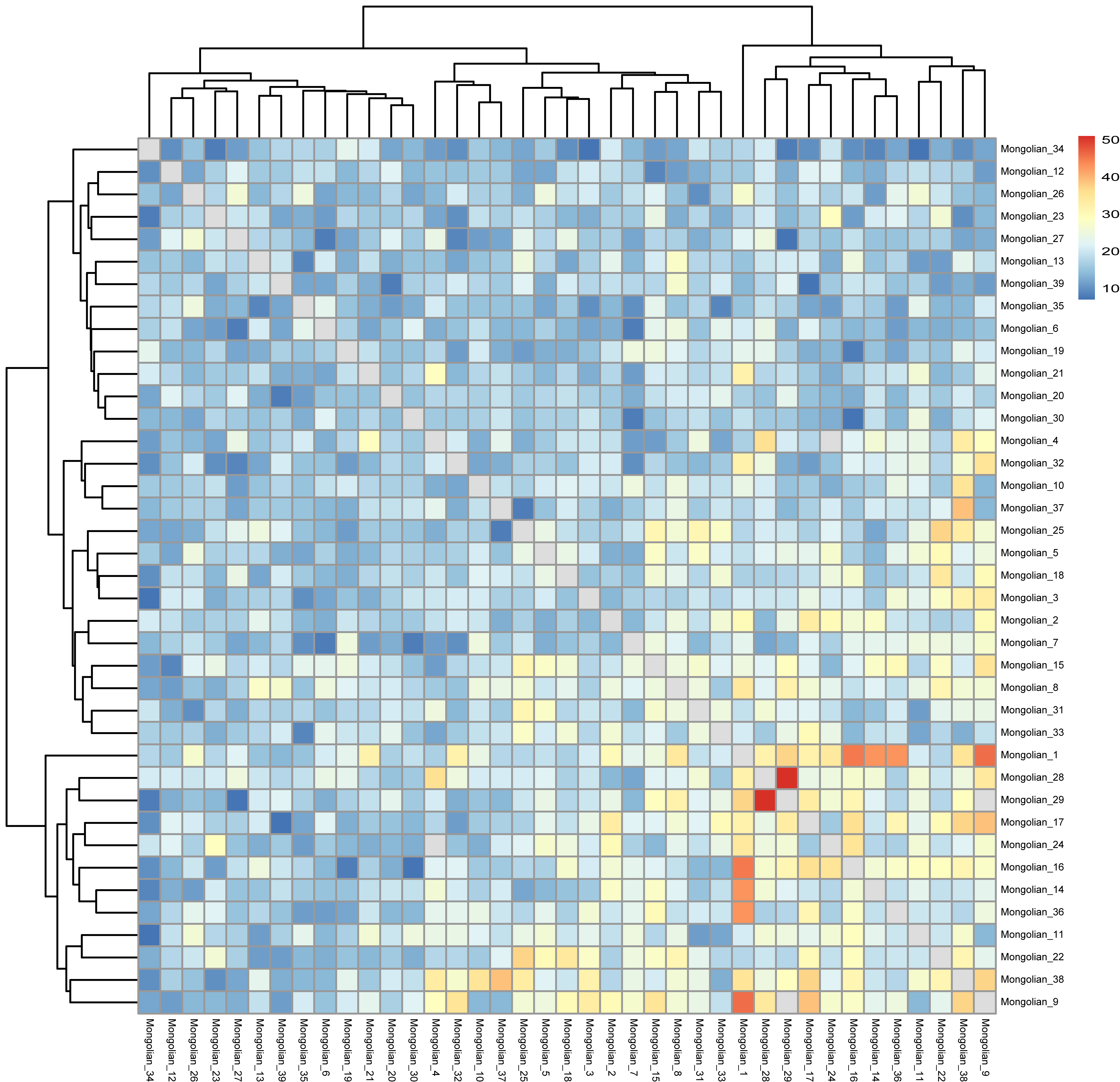

Supplement: Supplementary file 21 [file Image8.tif]

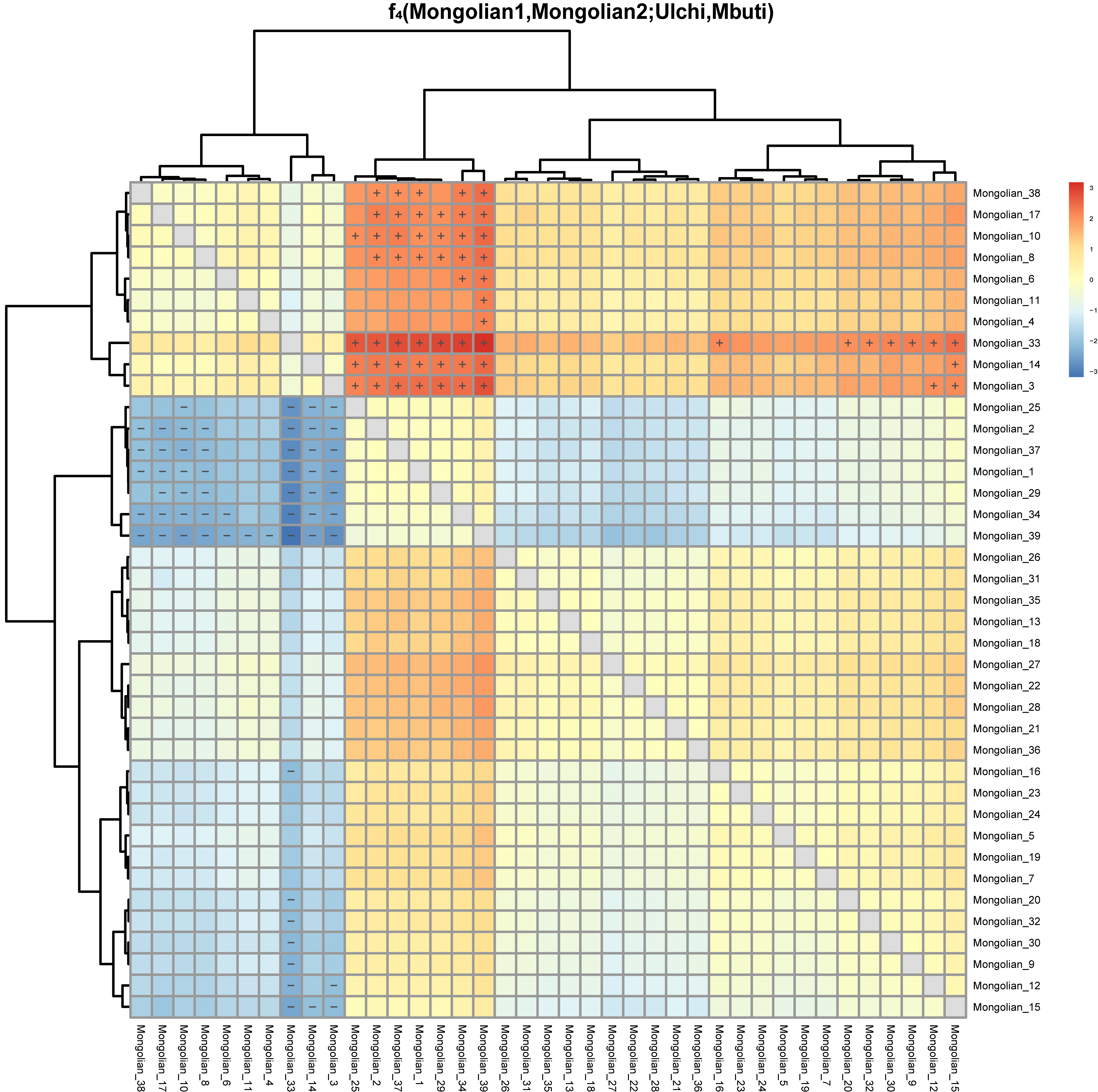

Supplement: Supplementary file 24 [file Image5.tif]
